# Biodegradation of poly(butylene succinate) in soil laboratory incubations assessed by stable carbon isotope labelling

**DOI:** 10.1038/s41467-022-33064-8

**Published:** 2022-09-28

**Authors:** Taylor F. Nelson, Rebekka Baumgartner, Madalina Jaggi, Stefano M. Bernasconi, Glauco Battagliarin, Carsten Sinkel, Andreas Künkel, Hans-Peter E. Kohler, Kristopher McNeill, Michael Sander

**Affiliations:** 1 Institute of Biogeochemistry and Pollutant Dynamics, ETH Zurich, 8092 Zurich, Switzerland; 2 Geological Institute, Department of Earth Sciences, ETH Zurich, 8092 Zurich, Switzerland; 3 BASF SE, Carl-Bosch-Strasse 38, Ludwigshafen, 67056 Germany; 4 Environmental Microbiology, Swiss Federal Institute of Aquatic Science and Technology (Eawag), 8600 Dübendorf, Switzerland

**Keywords:** Pollution remediation, Environmental impact

## Abstract

Using biodegradable instead of conventional plastics in agricultural applications promises to help overcome plastic pollution of agricultural soils. However, analytical limitations impede our understanding of plastic biodegradation in soils. Utilizing stable carbon isotope (^13^C-)labelled poly(butylene succinate) (PBS), a synthetic polyester, we herein present an analytical approach to continuously quantify PBS mineralization to ^13^CO_2_ during soil incubations and, thereafter, to determine non-mineralized PBS-derived ^13^C remaining in the soil. We demonstrate extensive PBS mineralization (65 % of added ^13^C) and a closed mass balance on PBS−^13^C over 425 days of incubation. Extraction of residual PBS from soils combined with kinetic modeling of the biodegradation data and results from monomer (i.e., butanediol and succinate) mineralization experiments suggest that PBS hydrolytic breakdown controlled the overall PBS biodegradation rate. Beyond PBS biodegradation in soil, the presented methodology is broadly applicable to investigate biodegradation of other biodegradable polymers in various receiving environments.

## Abstract (web summary)

This study applies stable carbon isotope labelling to study polymer biodegradation in soils. This labelling enables accurate and precise tracking of polymer carbon during biodegradation and, thereby, provides a holistic picture of this process.

## Introduction

Plastics composed of non-degradable polymers are major environmental pollutants of our time^1,2^. While best documented for marine^3–7^ and freshwater^8–10^ environments, plastic pollution also impacts terrestrial soil (eco)systems^11–13^. Among the latter, agricultural soils are particularly vulnerable to plastic pollution because they may receive plastic not only through littering and through the application of plastic-containing organic fertilizers such as sewage sludge and compost^13–17^, but also directly through the intentional use of plastics in agriculture^18–20^. The market of agricultural plastics is dominated by thin polymer films^21–24^, among which mulch films composed of polyethylene (PE) make up the largest share (i.e., 40%) with a predicted annual use of 3 million tons by 2021^21,24–26^. Mulch films are placed directly onto agricultural soils to increase crop yields through several means^24,27–29^. Yet, when these PE films are incompletely recovered from soils after use, persistent PE residues are left in soils and accumulate over time with repeated inputs. Incomplete recovery is particularly pronounced for mulch films with thicknesses of only a few µm, due to extensive weathering and embrittlement, and has led to concentrations of PE-residues in mulch-filmed soils as high as 500 kg ha^−1 ^^30^. Such accumulation of remnant plastic films not only lowers soil productivity and threatens food security^18,28^, but may also result in export of plastics from agricultural fields into adjacent (freshwater) ecosystems^11,13^.

A promising strategy to overcome accumulation of mulch film-derived plastics in soils is to use mulch films that are designed to biodegrade in soils. For mulch films, soil biodegradation is considered a viable end-of-life option given that re-collected films are often too contaminated with soil and plant debris to be efficiently recycled and too damaged to be reused^31^. Re-collected films would therefore require either landfilling or incineration, both of which are undesirable end-of-life treatment options in a circular economy. Furthermore, collection of used mulch films is labor-intensive and hence costly^32^. While soil biodegradable mulch films are already commercialized and show an increase in market share^27,29,31,33^, the process of polymer biodegradation in soils remains poorly understood, owing to limitations in analytical methods to study polymer biodegradation in soils.

Polymer biodegradation under oxic conditions, for instance in soils, describes the process by which microorganisms metabolically utilize the polymer carbon (i.e., C_polymer_) to yield energy under formation of carbon dioxide (C_mineralized_) and to build microbial biomass (C_biomass_). C_biomass_ is part of the soil organic matter pool (SOM) and herein is considered to include polymer-derived carbon present in living microbial cells as well as polymer-derived carbon in exudates from microbial cells and in microbial necromass. At any given time *t* during biodegradation, the mass balance on polymer carbon added at the onset of the experiment *t*_0_, C_polymer added_ (*t*_0_), is closed according to Eq. 1:^34–38^

1 $${{{{{{\rm{C}}}}}}}_{{{{{{\rm{polymer\; added}}}}}}}({t}_{0})={{{{{{\rm{C}}}}}}}_{{{{{{\rm{mineralized}}}}}}}(t)+{{{{{{\rm{C}}}}}}}_{{{{{\rm{biomass}}}}}}(t)+{{{{{{\rm{C}}}}}}}_{{{{{{\rm{polymer\; residual}}}}}}}(t)$$

where C_polymer residual_ (*t*) corresponds to carbon still present as polymer in the soil as long as biodegradation is incomplete. Combined, C_biomass_ (*t*) and C_polymer residual_ (*t*) make up the total non-mineralized polymer-derived carbon that is present in the soil at a given time *t*, C_non-mineralized_ (*t*):

2 $${{{{{{\rm{C}}}}}}}_{{{{{{\rm{non}}}}}}-{{{{{\rm{mineralized}}}}}}}(t)={{{{{{\rm{C}}}}}}}_{{{{{\rm{biomass}}}}}}(t){+{{{{{\rm{C}}}}}}}_{{{{{{\rm{polymer\; residual}}}}}}}(t)$$

According to Eqs. 1 and 2, assessing polymer biodegradation in soil incubations requires analytical methods not only for quantifying C_mineralized_ over the course of soil incubations but also for quantifying C_non-mineralized_ as well as its relative contributions from C_polymer residual_ and C_biomass_.

Previous soil incubation studies using non-labelled polymers followed mineralization through respirometric measurements of the excess amount of CO_2_ formed in soils with added polymer relative to polymer-free control soils^37,39–41^. Yet, accurately quantifying this excess CO_2_ amount is challenging, particularly when polymers mineralize slowly and when soils show comparatively large background CO_2_ formation from SOM mineralization^42^. Furthermore, these respirometric analyses are susceptible to an artefact that results when polymer addition to soils increases or decreases background SOM mineralization rates. Such positive or negative priming effects in soils are well-documented for a range of diverse substrates and imply that polymer-free controls may no longer capture background SOM mineralization^43–45^. Besides these challenges in respirometric measurements, analytical methods for quantifying C_non-mineralized_ and C_polymer residual_ in soils are missing until now. As a consequence, authors of past studies could not assess polymer carbon mass balances during soil incubations—a prerequisite to accurately quantify polymer biodegradation^46^—nor determine the relative contribution of C_polymer residual_ and C_biomass_ to C_non-mineralized_. The latter is important, however, as formation of C_biomass_ is a desired biodegradation outcome whereas residual polymer carbon, C_polymer residual_, implies that polymer biodegradation is incomplete at the time of the soil analysis.

The above analytical challenges to assess polymer biodegradation in soils can be countered by using carbon-isotope labelled polymers and by tracking the labelled carbon during the soil incubations^47^. In past work, researchers have successfully used ^14^C-labelled polymers for studying transformation of polymers during soil incubations^48,49^. However, ^14^C-labelling requires one to work under radioisotope-specific safety regulations and with laboratory infrastructure and instrumentation solely designated to radioisotope analyses^40,50^. A preferable alternative is the use of ^13^C-labelled polymers in incubation studies combined with ^13^C-selective analytical techniques^26,42,47,51^. In a previous study, we incubated ^13^C-labelled poly(butylene adipate-*co*-terephthalate) (PBAT) in a soil and used isotope sensitive cavity ring down spectroscopy for tracking formation of ^13^CO_2_ from PBAT−^13^C as well as element-specific, isotope-selective nanoscale secondary ion mass spectrometry (NanoSIMS) for demonstrating incorporation of PBAT−^13^C into microbial biomass^52^. However, we followed PBAT mineralization only over a comparatively short soil incubation time of 42 days and we did not attempt to quantify C_non-mineralized_ and C_polymer residual_ at the end of the incubations.

In this work, we present extensive data on polymer biodegradation during long-term soil incubations by employing an analytical workflow based on stable-carbon isotope (^13^C) labelled synthetic polymers as substrates. We continuously monitor polymer mineralization into ^13^CO_2_ over the course of the incubations and quantify both C_non-mineralized_ and C_polymer residual_ in the soils after terminating the incubations. We use poly(butylene succinate) (PBS) as a model biodegradable polymer because of its commercial relevance as biodegradable material in agricultural films and because it serves as a model for other synthetic soil biodegradable polyesters composed of two monomers^53,54^. More specifically, we use three variants of PBS that are monomer- and position-specifically ^13^C-labelled (i.e., PB(1,4−^13^C_2_-S) and PB(2,3−^13^C_2_-S) and P(^13^C_4_-B)S). We complement the PBS soil biodegradation studies with incubations of only the labelled monomers 1,4- and 2,3-^13^C_2_-succinate (S) and ^13^C_4_-butanediol (B) in the same soil to assess differences in the mineralization dynamics of the monomers and the corresponding PBS variants^39^. Furthermore, we follow mineralization of ^13^C-cellulose in the same soil, because this biopolymer is often used as a positive biodegradation control in polymer biodegradation studies and standards^55^. Kinetic modeling of the experimental data highlights the importance of assessing the relative importance of C_biomass_ and C_polymer residual_ for accurate data interpretation. This work showcases advances in process understanding of polymer biodegradation in soils that result from using stable carbon isotope labelling.

## Results

### Mineralization of ^13^C-labelled PBS variants and monomers during soil incubations

**Fig. 1:**
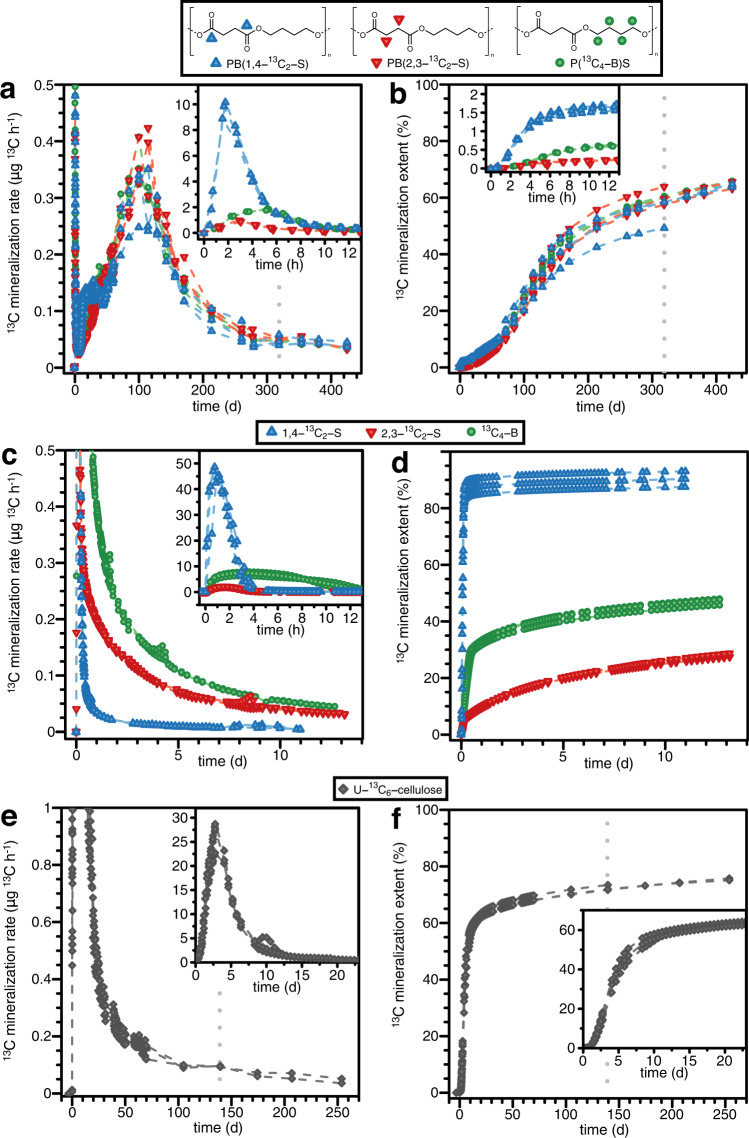
Mineralization of ^13^C-labelled poly(butylene succinate), monomers and cellulose during soil incubations. Measured mineralization rates (**a**, **c**, **e**) and resulting calculated cumulative mineralization extents (i.e., C_mineralized_; **b**, **d**, **f**) for the three variants of ^13^C-labelled poly(butylene succinate) (PBS) (i.e., PB(1,4-^13^C_2_-S), blue up-triangles); PB(2,3-^13^C_2_-S), red down-triangles); and P(^13^C_4_-B)S, green circles — **a**, **b**), the corresponding ^13^C-labelled succinate and butanediol monomers (i.e., 1,4-^13^C_2_-S, blue up-triangles; 2,3-^13^C_2_-S, red down-triangles; and ^13^C_4_-B, green circles — **c**, **d**), and U-^13^C_6_-cellulose (grey diamonds — **e**, **f**), during soil incubations. The ordinate units for the insets in **a**–**c**, **e**, **f** are identical to those of the ordinate units of the respective main panels. Dashed lines between measurement data points are linear interpolations and serve to guide the eye. Data points are shown for individual incubation bottles, corresponding to triplicate incubations up to 319 days for the three PBS variants and 139 days for cellulose (the timepoints are indicated by vertical grey dotted lines, **a**, **b**, **e**, **f**; at these times, one of the respective triplicate flasks was removed from the incubation system and the soil in this flask was processed for further analysis) or duplicate incubations from 319 to 425 days for PBS variants and from 139 to 254 days for cellulose. Each labelled monomer was incubated in triplicates for a total time of 14 days. Mineralization data of the monomer ^13^C_4_-B (**d**) up to four days of incubation was previously published as supplementary information in Zumstein et al.^52^.

Within less than one hour of adding PBS to the soils we detected PBS-derived ^13^CO_2_ in the efflux gas of the incubation bottles. The rates of ^13^CO_2_ formation reached a first maximum after 2–5 hours of incubation (inset in Fig. 1a). These maximum mineralization rates were variant-specific and decreased in the order of PB(1,4-^13^C_2_-S) > P(^13^C_4_-B)S > PB(2,3-^13^C_2_-S) from approximately 10 to 1 μg ^13^C h^-1^ after 2 h of incubation. Following these initial maxima, mineralization rates of all three variants continuously decreased to very low values after 1 week of incubation. At this time, C_mineralized_ corresponded to 2.1, 1.1, and 0.5% of the added PBS-^13^C for PB(1,4-^13^C_2_-S), P(^13^C_4_-B)S and PB(2,3-^13^C_2_-S), respectively (inset in Fig. 1b for the first 12 h). Starting from day 7 of incubations, mineralization rates of the three PBS variants increased again slowly and reached second maxima (i.e., 0.31–0.36 μg ^13^C h^-1^) at approximately day 100 of the incubations (Fig. 1a). At this time, PBS variant-averaged C_mineralized_ corresponded to between 21 and 24% of the added ^13^C (Fig. 1b). The mineralization rates subsequently decreased continuously over the remaining duration of the soil incubations to very low final rates. While rates and cumulative extents of mineralization were different for the three PBS variants during the first 50 to 70 days of the incubations (see Supplementary Note 1), mineralization rates and extents of the three variants became indistinguishable at longer incubation times. We terminated the incubations for one of the triplicate flasks of each variant after 319 days to verify closed mass balances on PBS-^13^C at this time and to be able to extract residual PBS at an intermediate incubation time (see below; dotted vertical lines in Fig. 1a, b represent the times at which these incubations were terminated). We continued incubating the remaining two flasks per variant for a total of 425 days when mineralization rates had decreased to low values and C_mineralized_ had reached between 59 ± 4 % and 65 ± 1 % of the PBS-added ^13^C (Fig. 1b). The final mineralization extents of the three PBS variants were statistically indistinguishable (by one-way ANOVA test, not significant at the α error level 0.01), implying that the labelled carbon in PBS mineralized to similar final extents irrespective of the labelling position. While mineralization of PBS-^13^C added to soil was incomplete at the end of the incubations (i.e., C_mineralized_ < 100 %), mineralization was still progressing at that time. Consequently, mineralization extents of PBS would have increased to higher final values if we had continued the soil incubations beyond 425 days.

As compared to the three PBS variants, the corresponding three labelled monomers showed much faster mineralization (Fig. 1c, d). This finding strongly suggests that mineralization rates of PBS in soils were controlled by enzymatic PBS breakdown to low molecular weight hydrolysis products, while their subsequent microbial uptake and utilization was comparatively fast. In addition to having higher mineralization rates than PBS, the monomers showed both monomer- and carbon position-specific mineralization: maximum rates and final extents of mineralization at 14 days of incubation decreased in order of 1,4-^13^C_2_-S > ^13^C_4_-B > 2,3-^13^C_2_-S (rates in Fig. 1c and extents in Fig. 1d; see Supplementary Note 2 for rate data normalized to the maximum value for each bottle). We note that the order in the mineralization rate of the respective PBS variants during the first hours of soil incubation mirrored this order in the mineralization rates of the monomers (insets Fig. 1a, b for the first 12 hours of incubation).

For the following reasons, we ascribe initial high PBS mineralization rates during the first week of incubation to the microbial utilization of low molecular weight mono- and oligomers that were present in the lab-scale synthesized PBS and that readily diffused out of the PBS into the soils during the first day of the incubations. First, the rapid initial onset of ^13^CO_2_ formation suggests that ^13^C-containing compounds were immediately available to soil microorganisms before microbial degraders colonized the PBS surfaces and started to enzymatically hydrolyze the PBS. Second, C_mineralized_ at the end of this initial phase was only very small (i.e., <2% of PBS-added ^13^C; inset Fig. 1b) and in good agreement with reported low percent amounts of non-polymerized monomers and short oligomers in synthetic polyesters^56^. Third, the initial mineralization in incubations of the three PBS variants and in incubations of the corresponding monomers showed the same pattern of position- and monomer-specificity, supporting that microorganisms utilized monomeric B and S and low molecular weight BS oligomers that were present in the PBS and diffused out into the soil solution during the first hours to days after PBS addition. We note that the variant specificity of succinate can be rationalized based on its cellular metabolism. The higher rates of ^13^CO_2_ formation for PB(1,4-^13^C_2_-S) and 1,4-^13^C_2_-S than for PB(2,3-^13^C_2_-S) and 2,3-^13^C_2_-S likely resulted from preferential catabolic decarboxylation of the 1,4-carbons of S, whereas the 2,3-carbons of S were preferentially used in anabolism: for succinate molecules that enter the citric acid cycle of aerobic microorganisms, one of the 1- and 4-carbons (carboxylate groups) is converted to CO_2_ during a single cycle whereas the 2- and 3-carbons of succinate remain in the citric acid cycle during the first cycle and thus are expected to be used preferentially for the synthesis of biomolecules (see Supplementary Note 3 for a more detailed discussion). The mineralization rates and extents of ^13^C atoms from uniformly ^13^C-labelled butanediol were intermediate to those in 1,4-^13^C_2_-S and 2,3-^13^C_2_-S, suggesting that one or both alcohol groups in butanediol were oxidized to carboxylates followed by decarboxylation to CO_2_ and incorporation into microbial biomass. The position- and monomer-specificity observed for PBS mineralization during the first week of incubation therefore suggests that carbons in the 2- and 3-positions of both S- and B-containing substrates that diffused out of bulk PBS were preferentially incorporated into microbial biomass.

We assign the second, prolonged phase of ^13^CO_2_ formation from PBS between 7 and 425 days of soil incubation to the mineralization of bulk PBS. Mineralization rates were likely controlled by hydrolytic breakdown of the PBS, presumably catalyzed by microbial extracellular esterases given that abiotic hydrolysis of PBS is slow^57,58^. Hydrolytic breakdown as the rate determining step in PBS biodegradation is supported by the much slower mineralization of PBS than of monomers B and S directly added to soils (Fig. 1c, d). Furthermore, slow PBS hydrolysis can explain the indistinguishable cumulative mineralization extents of the three different PBS variants from approximately 70 days of incubation onward while mineralization of the respective labelled B and S was monomer- and position-specific. The absence of position specificity of carbon utilization during this second phase points at microorganisms using PBS-derived carbon for cell maintenance with limited incorporation into microbial biomass. Low incorporation into biomass may have been only apparent if PBS-derived ^13^C incorporated into biomass was re-mineralized at rates higher than those at which PBS-^13^C became available to the microbial cells and was incorporated into the biomass. Extensive conversion of all PBS-derived carbon into CO_2_ towards the end of the incubation is supported by the finding that C_non-mineralized_ remaining in the soils at the end of the incubations was present primarily as residual PBS, C_polymer residual_, and not as microbial biomass, C_biomass_ (see below for data, modeling of data, and discussion). We discuss possible explanations for the decrease in PBS mineralization rates after 100 days of incubation in more detail below after presenting data on extracted residual PBS in the soils at the end of the incubations and kinetic modeling of the experimental data.

### Mineralization of ^13^C-labelled cellulose during soil incubations

Mineralization of the reference material cellulose started within a few hours after its addition to the soil (Fig. 1e). As compared to PBS, cellulose mineralization rates did not show an initial maximum during the first hours of incubation. Instead, mineralization rates continuously increased from the onset of the incubation to maximum values of 26 ± 3 μg ^13^C h^-1^ after about three days of incubation, at which time approximately 17 ± 2% of the added cellulose-^13^C had already mineralized (Fig. 1f). Cellulose mineralization rates subsequently decreased to 0.58 ± 0.07 μg ^13^C h^-1^ after 20 days of incubation, at which time 62 ± 1% of the added cellulose-^13^C had mineralized. At this point, the cumulative mineralization curve shows a distinct decrease in slope and therefore a transition from an initial phase of faster to a subsequent phase of slower mineralization (Fig. 1e, f). Over the remaining 234 days of incubation, the mineralization rates continuously decreased to very small final values at day 254 when we terminated the incubations. The final extent of cellulose mineralization was 75 ± 1% at day 254 (mean ± deviation from the mean of duplicate incubations, as we removed one incubation flasks at 139 days). Cellulose mineralization extents after 90 days of soil incubation were approximately 70 ± 1 % and in good agreement with the range of cellulose mineralization extents of 54–78% after 90 days of soil incubation previously reported in the literature^59,60^.

Overall faster mineralization of cellulose than that of PBS suggests a high abundance and activity of microorganisms secreting extracellular cellulases in the tested soil. Cellulose incubations only showed one maximum in mineralization rates as opposed to incubations of the PBS variants which showed initial peaks of high mineralization rates that preceded second peaks with lower maximum mineralization rates. This finding implies either that the tested cellulose did not release low-molecular weight constituents or that the mineralization of bulk cellulose was sufficiently fast from the onset of the incubation to mask mineralization of low molecular weight constituents that were present in the added cellulose and released into the soil. We ascribe the increase in mineralization rates over the first 5 days of incubation to an adaptation of soil microbes to the presence of cellulose as substrate. This adaptation likely involved both colonization of the cellulose fiber surfaces and upregulation and increased secretion of cellulases^61^. The transition from higher to lower cellulose mineralization rates at day 20 day of the incubation likely reflects complete depletion of cellulose in the soil and thus a shift in the primary source of formed ^13^CO_2_ from cellulose to the slower turnover of microbial biomass that had incorporated cellulose-^13^C during the first 20 days of incubation. This interpretation is consistent with past cellulose mineralization studies^59,60,62,63^, as well as with our results of data modeling (see below).

### Quantification of non-mineralized PBS-added ^13^C remaining in soil

The use of ^13^C-labelled PBS allowed us to selectively quantify not only PBS mineralization, C_mineralized_, but also the total amount of PBS-added ^13^C that remained in the soil at the end of the incubations, C_non-mineralized_ (Eq. 2). In an initial attempt to determine C_non-mineralized_, we freeze-dried and milled the soil from the incubation flasks and took 10 mg aliquots for elemental analysis (EA) coupled to isotope ratio mass spectrometry (IRMS) to quantify PBS- (and cellulose-) derived ^13^CO_2_. We note that the EA-IRMS approach was technically restricted to 10 mg soil aliquots. Despite all PBS incubations bottles showing comparable final C_mineralized_ (Fig. 1b), these initial EA-IRMS analyses yielded very high variations in C_non-mineralized_, and incomplete mass balances (i.e., 86 ± 7% mass balance on added PBS-^13^C; *n* = 3 replicate incubation bottles). We ascribed these large variations to residual PBS being present as particles that were not representatively subsampled in the 10 mg soil aliquots for EA-IRMS.

**Fig. 2:**
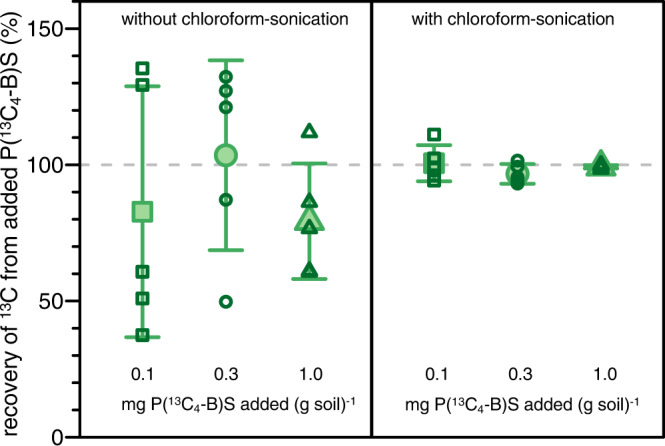
Effect of soil treatment on quantified amounts of non-mineralized poly(butylene succinate) (PBS) remaining in soil. Recovery of butanediol-^13^C-labelled PBS (i.e., P(^13^C_4_-B)S) from soils containing known added amounts (i.e., 0.1 (squares), 0.3 (circles), and 1.0 (triangles) mg P(^13^C_4_-B)S per g soil) without (left) and with (right) chloroform-sonication treating the soils prior to EA-IRMS on small soil aliquots of 10 mg. Recoveries are expressed as amount of ^13^C quantified in percent of the expected ^13^C amount if added PBS particles were representatively sampled in the small soil aliquots for EA-IRMS. The open symbols depict recoveries of five individually analyzed replicates per soil, and closed symbols and corresponding error bars depict the average and standard deviations of the five replicates, respectively. Recovery of PBS-derived ^13^C was inaccurate and imprecise when we analyzed aliquots taken from soils that were only sieved and milled (left side). Conversely, recovery was complete, accurate and precise when analyzing subsamples from sieved and milled soils that were additionally treated by chloroform-sonication (right side).

To overcome non-representative soil subsampling for particulate PBS, we developed a chloroform-sonication soil treatment procedure. For validation of the treatment procedure, we added P(^13^C_4_-B)S to three samples of the same soil used in the incubations to result in concentrations of 0.1, 0.3, and 1.0 mg PBS per g of soil. Following freeze-drying, sieving, and milling these three soil samples (see Methods Section), EA-IRMS analysis of 10 mg soil subsamples resulted in measured PBS concentrations that were both inaccurate and imprecise (Fig. 2, left side), confirming that 10 mg soil subsamples were too small to representatively quantify particulate PBS in the soil. We subsequently added chloroform to another three dried and milled soil samples with added P(^13^C_4_-B)S to dissolve the PBS particles and sonicated these samples to disperse the then dissolved PBS. Following removal of chloroform through evaporation, EA-IRMS analysis of 10 mg soil subsamples resulted in both accurate and precise quantification of the added PBS-^13^C in the soils (Fig. 2, right side; recoveries of 100 ± 7, 97 ± 4, and 99 ± 1% of the PBS-added ^13^C for soil samples with 0.1, 0.3, and 1.0 mg PBS g^-1^ soil, respectively). While not explicitly tested, we assume that this chloroform-sonication treatment would also lyse microbial cells and disperse released cell constituents in the soil, thereby also ensuring that 10 mg soil aliquots representatively sample for PBS-derived ^13^C incorporated into microbial biomass. Further details on the homogenization procedure and results from additional analyses are provided in the Supplementary Note 4.

**Fig. 3:**
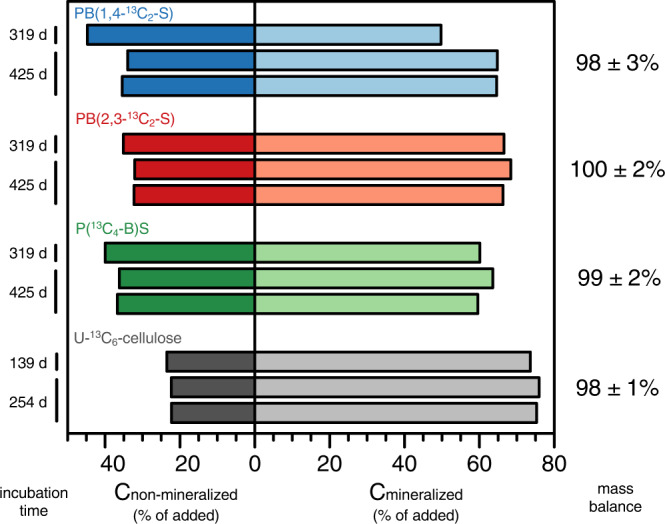
Mass balance on poly(butylene succinate) (PBS)- and cellulose-added ^13^C over the soil incubations. Left side (darker shaded bars): total non-mineralized PBS- and cellulose-derived ^13^C (i.e., C_non-mineralized_) in the soils at the end of the incubations shown for the three ^13^C-labelled PBS variants (i.e., PB(1,4-^13^C_2_-S), PB(2,3-^13^C_2_-S), and P(^13^C_4_-B)S; blue, red and green bars, respectively) and for ^13^C-labelled cellulose (grey bars). Right side (lighter shaded bars): final cumulative amounts of PBS- and cellulose-^13^C that had mineralized over the course of the soil incubations (replotted from Fig. 1b, f, respectively). Both C_non-mineralized_ and C_mineralized_ are expressed as percent of the amounts of PBS-^13^C and cellulose-^13^C that we initially added to the soils at the onset of the incubations. The values to the right of the plot correspond to the mass balance on added ^13^C (i.e., the sum of C_non-mineralized_ and C_mineralized_ in percent of the amount of PBS-^13^C and cellulose-^13^C that we initially added to the soils at the onset of the incubations (quantified from results of all triplicate incubation bottles)). Incubations of PBS and cellulose were run in triplicates up to 319 and 139 days and in duplicates from 319 to 425 days and from 139 to 254 days, respectively.

We subsequently applied the chloroform-sonication treatment described above to the milled soils from the PBS incubation experiments, followed by EA-IRMS analysis of 10 mg aliquots from the treated soil (see Methods section for details). For PBS soil incubation bottles removed from the mineralization setup after 319 and 425 days, C_non-mineralized_ corresponded to 40 ± 5% (*n* = 3) and 34 ± 2% (*n* = 6) of the initially added PBS-^13^C, respectively. Consistent with the mineralization data, the ^13^C remaining in the soils decreased with increasing incubation time. More importantly, the summed C_non-mineralized_ and C_mineralized_ for PBS were in excellent agreement with C_polymer_ initially added to soils (Fig. 3, right column, with average recoveries of 98 ± 3, 100 ± 2, and 99 ± 2 % of PBS-added ^13^C for PB(1,4-^13^C_2_-S), PB(2,3-^13^C_2_-S), and P(^13^C_4_-B)S, respectively). The mass balance of PBS-added ^13^C was therefore closed over all long-term soil incubations, demonstrating accurate tracking of ^13^C-labelled PBS into both C_mineralized_ and C_non-mineralized_ over the course of the biodegradation process.

### Quantification of non-mineralized cellulose-added ^13^C remaining in soil

Similar to PBS incubations, we freeze-dried, sieved and milled the soils from cellulose incubation bottles. Direct EA-IRMS analyses of aliquots from the milled soils yielded C_non-mineralized_ of 23.5% of the initially added cellulose-^13^C in one incubation bottle after 139 days, and of 22.3% and 22.2% in the remaining two bottles after 254 days of incubation. As shown above for PBS, the mass balances on cellulose carbon during its biodegradation in soil were also closed: the sum of C_non-mineralized_ and C_mineralized_ corresponded to 98 ± 1% (*n* = 3) of the cellulose-^13^C initially added to the soils. Our finding that direct EA-IRMS analysis of cellulose-derived ^13^C in the soils was accurate and precise implied that soil milling was sufficient to result in representative subsampling of non-mineralized cellulose-derived ^13^C in 10 mg soil aliquots for EA-IRMS. The finding that no additional treatment besides milling was needed to accurately quantify C_non-mineralized_ indicates a uniform distribution of cellulose-derived ^13^C in the soil. This uniform distribution likely resulted from having added cellulose to the soils as a very fine powder and from the non-mineralized ^13^C presumably being present in microbial biomass rather than residual cellulose. However, the latter two pools could not be clearly distinguished as a methodology to extract and quantify cellulose from soils is missing.

### Quantification of residual PBS in soil

We determined the relative contribution of residual PBS (C_polymer residual_) to the overall non-mineralized PBS-derived carbon remaining in the soils, C_non-mineralized_, by Soxhlet extraction of residual PBS from the soils followed by the quantification of PBS in the soil extracts using quantitative ^1^H NMR. We confirmed complete extraction and quantification of PBS from the tested soil in separate spike-recovery experiments of PBS in the same soil that we had used in the incubation (see Supplementary Note 5 for details). We note that the quantification of C_polymer residual_ was restricted to PBS because cellulose does not readily dissolve in solvents compatible with Soxhlet extractions.

**Fig. 4:**
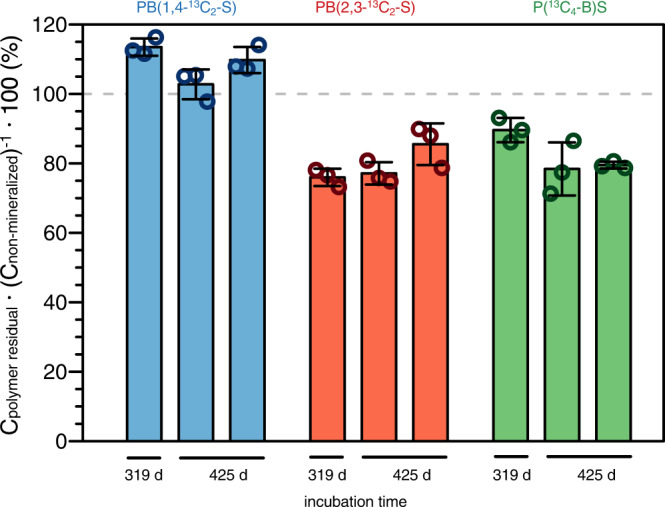
Residual amounts of poly(butylene succinate) (PBS) extracted from soils at the end of the incubations. Amounts of PBS extracted from soils (i.e., C_polymer residual_) expressed in percent of the total non-mineralized amount of PBS-added ^13^C (i.e., C_non-mineralized_) remaining in the soils at the end of the incubations. Results are shown for triplicate incubations of the three ^13^C-labelled PBS variants (i.e., PB(1,4-^13^C_2_-S), PB(2,3-^13^C_2_-S), and P(^13^C_4_-B)S, shown in blue, red, and green, respectively). For each soil from the incubations, residual PBS was quantified in three soil subsamples; open symbols represent results from each individual extracted subsample, while colored bars and error bars depict the mean ± one standard deviation of these triplicate extractions.

After 425 days of incubation, C_polymer residual_ corresponded to 37 ± 2%, 26 ± 1%, and 29 ± 1% of the initially added PB(1,4-^13^C_2_-S), PB(2,3-^13^C_2_-S), and P(^13^C_4_-B)S, respectively. More importantly, comparing C_polymer residual_ to C_non-mineralized_ revealed that the remaining extracted PBS accounted for most or all of the total PBS-derived ^13^C that was not mineralized (Fig. 4). We ascribe the higher apparent contribution of remaining PB(1,4-^13^C_2_-S) to C_non-mineralized_ as compared to P(^13^C_4_-B)S or PB(2,3-^13^C_2_-S) to the preferential mineralization of the carboxyl carbons in PB(1,4-^13^C_2_-S) and therefore smaller incorporation of ^13^C from PB(1,4-^13^C_2_-S) into the microbial biomass, as detailed above for monomer mineralization experiments. Overall, the extracted PBS amounts imply that the amount of PBS-derived carbon incorporated into biomass or SOM (C_biomass_) was approximately 7 ± 2% of the originally added PBS carbon. The comparatively small C_biomass_ pool at the end of the incubation implies that soil microorganisms used PBS-derived carbon primarily for energy production and cell maintenance with limited incorporation into microbial biomass. Low incorporation into biomass may have resulted from a low carbon use efficiency or, if PBS-derived ^13^C was incorporated into biomass, that the rates of ^13^C incorporation were smaller than the rates at which ^1^^3^C incorporated in the biomass was re-mineralized to ^13^CO_2_. We conclude that the continuous slow mineralization determined at the end of the soil incubations resulted primarily from the mineralization of residual PBS.

### Interpretation and modeling of PBS and cellulose biodegradation dynamics

Using ^13^C-labelled PBS as an added substrate, along with ^13^C-selective analytical techniques in soil incubation experiments, allowed us to establish mass balances on PBS-^13^C over long-term soil incubations and to unequivocally demonstrate that PBS biodegraded extensively in the tested soil (see Supplementary Note 6 for a summary of biodegradation results for all PBS- and cellulose-containing incubations). The soil, therefore, contained microorganisms capable of metabolically utilizing PBS breakdown products, likely formed through hydrolysis of backbone ester bonds, for energy production and the formation of microbial biomass. Soil extractions revealed that residual PBS made up most of C_non-mineralized_ at the end of the incubations. We ascribe the apparently low incorporation of PBS-derived carbon into microbial biomass to this carbon cycling through the microbial biomass pool at rates higher than the rates at which this carbon became available to the microorganisms through enzymatic PBS hydrolysis. This interpretation is supported by the finding that mineralization of bulk PBS was much slower than of the corresponding ^13^C-labelled PBS monomers in the same soil: PBS hydrolytic breakdown into labile carbon rather than microbial utilization of this carbon therefore controlled overall rates of PBS biodegradation in the tested soils. Finally, while PBS biodegradation was incomplete at the end of the incubations, progressing mineralization at that time implies that PBS mineralization would increase to higher final extents if we had continued the incubations.

Quantifying residual PBS in the soil proved essential for the correct interpretation of the biodegradation process: most of the non-mineralized PBS-^13^C was present in the form of PBS while the incorporation extent of PBS-derived ^13^C into microbial biomass was comparatively small. Without data on residual PBS, an alternative—but incorrect—interpretation of the mineralization data would have been that the non-mineralized PBS-^13^C remaining in the soil at the end of the incubation was extensively incorporated into microbial biomass from which the ^13^C subsequently was only slowly mineralized (see modeling section below). The decrease in PBS mineralization rates after 100 days of incubation in combination with residual PBS being present throughout the incubation strongly suggests that at least one system factor started to constrain overall PBS biodegradation after about three months into the incubations. We address potential factors in the subsequent discussion.

**Fig. 5:**
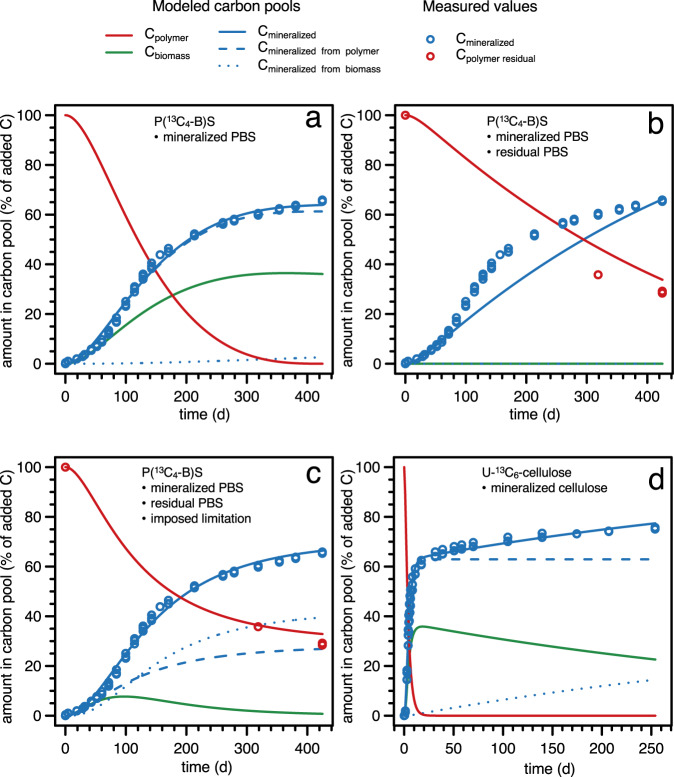
Kinetic modeling of poly(butylene succinate) (PBS) and cellulose biodegradation data. Experimental data for PBS labelled in butanediol (i.e., (P(^13^C_4_-B)S; **a**–**c**) (open symbols in blue for mineralization and red for extracted PBS) and for cellulose (**d**; open symbols in blue for mineralization) is replotted from figures above. P(^13^C_4_-B)S model details and data are also given in the Supplementary Note 7, along with model details and data of the other ^13^C-labelled PBS variants. The solid, dashed, and dotted lines represent the modeled carbon pools as specified in the figure legend. For PBS, three different model types were run: a first model which only fitted the mineralization data, C_mineralized_ (**a**), a second model which fitted  C_mineralized_ as well as the extracted PBS from the soil, C_polymer residual_,  assuming a constant, first order PBS hydrolytic breakdown rate constant (**b**) and a third model which fitted both C_mineralized_ and C_polymer residual_ and in which we allowed for a decrease in the effective PBS hydrolytic breakdown rate constant over the course of the incubation (**c**). We only fitted the first model to the cellulose mineralization data, C_mineralized_ (**d**), as detailed in the text.

To support our interpretation of PBS biodegradation dynamics, we modeled the PBS mineralization and extraction data using a basic carbon flux model. The modeling aimed at substantiating the importance of PBS extraction data for correct process interpretation as well as supporting our qualitative interpretation that PBS biodegradation slowed down beyond 100 days of incubation. We developed a fit-for-purpose model that allowed for flow of PBS carbon via its monomeric and oligomeric hydrolysis products (i.e., a labile carbon pool) into microbial cells. Inside the cells, the carbon use efficiency (CUE) defined the fraction of the PBS-derived carbon that was directly mineralized to CO_2_ (catabolism) vs. incorporated into microbial biomass (anabolism) (CUE = fraction of total carbon taken up that is used for biomass formation). The flow of carbon between all the pools was modeled assuming first-order kinetics. While PBS biodegradation is a microbial process, we decided against using an explicit microbially-driven model with cell density-dependent turnover of PBS carbon^64,65^, because we have no direct data for changes in C_biomass_ over the course of the incubation and because C_biomass_ was small at the end of the incubations. For the modeling of PBS data described below, we present data for the variant P(^13^C_4_-B)S in Fig. 5. We obtained similar modeling results for the other variants, shown in the Supplementary Note 7.

Modeling of only the PBS mineralization data, C_mineralized_—excluding the data on residual PBS, C_polymer residual_, that we obtained by soil extraction—resulted in a false model output of complete PBS biodegradation after 360 (±50) days of soil incubation (i.e., >99% conversion of the added PBS to C_mineralized_ and C_biomass_ with no residual PBS) and that a large fraction of the PBS-derived carbon was incorporated into microbial biomass, C_biomass_ (fitted CUE = 0.34 ± 0.06) (Fig. 5a). In this model fit, the decrease in overall mineralization rates beyond 100 days of soil incubation is ascribed falsely to slow cycling of PBS-derived ^13^C through a large microbial biomass pool. The overall high quality of the data fit in combination with a reasonable fitted CUE value (the theoretical maximum CUE for microbial growth is about 0.6)^66^ highlights the necessity to include C_residual polymer_ data to correctly describe the biodegradation process.

We subsequently altered the model by including the experimental C_polymer residual_ values that we obtained by extraction of the soils at the end of the incubations. While there were only three C_polymer residual_ values for each PBS variant, these data put a constraint on the amount of residual PBS material and, thereby, ensured that biodegradation dynamics could be accurately modeled. As before, we allowed the model to fit only a single rate constant for PBS hydrolytic breakdown. The model fit resulted in much lower apparent CUEs (i.e., CUE < 0.001) for PBS-derived carbon and thus primarily ascribed ^13^CO_2_ formation throughout the incubation to direct PBS mineralization, i.e. no incorporation of PBS-derived carbon into the C_biomass_ pool (Fig. 5b). However, this model did not adequately fit the sigmoidal shape of the experimental mineralization data beyond approximately 100 days of incubation.

In a final model modification, we fitted both C_mineralized_ and C_polymer residual_ but allowed for a decrease in the effective PBS hydrolytic breakdown rate constant over time using an exponential decay function (see Methods section for details). This modified model resulted in good data fits for both C_mineralized_ and C_polymer residual_ (Fig. 5c; shown for a model run with CUE = 0.4). Three outcomes of fits from this model are noteworthy. First, the model fitted an increase in C_biomass_ up to approximately 100 days of soil incubation, in good agreement with the timeframe over which we observed differences in the extents of mineralization between the three PBS variants and thus position-specific incorporation of PBS-derived carbon into microbial biomass (Supplementary Note 1). The modeled decrease in C_biomass_ after approximately 100 days of incubation implies that rates of PBS-derived carbon cycling out of the microbial biomass into CO_2_ were faster than the rates at which PBS-derived carbon was incorporated into the biomass and hence supplied to the cells through enzymatic PBS hydrolysis. This modeling result is consistent with kinetic masking as the explanation for the apparent loss of position-specific carbon incorporation into microbial biomass from the three PBS variants beyond 100 days of incubation. Second, the overall quality of the model fits was little affected by the maximum CUE values with which we constrained the model and to which the model fit converged (see Supplementary Note 7). This finding implies that CUE values were not accurately fitted, reflecting that modeled rates of biomass mineralization exceeded rates at which PBS-derived carbon became metabolically available to the cells beyond approximately 100 days of incubation, independent of which CUE was used. Third, the improvement in the quality of the model fit when allowing for a decrease in the effective PBS depolymerization rate constant supported the presence of at least one system factor that started to limit PBS biodegradation after about 100 days during long-term soil incubations.

Identifying factors that constrained PBS biodegradation is challenging because we did not design the incubation experiments to test for specific factors. Yet, several factors are plausible. One explanation is that a fraction of the PBS particles added to the soils were transferred to microenvironments that were physically inaccessible to microbial degraders. Another explanation is that microcrystalline domains in PBS became enriched in the residual PBS over time as biodegradation continued, possibly also involving re-crystallization reactions as biodegradation progressed^67,68^. Because microcrystalline domains undergo slower enzymatic hydrolysis than amorphous domains, their enrichment is expected to slow down PBS mineralization. However, these explanations would call for a non-accessible fraction (due to the physical environment or PBS crystallinity) of as much as 40% of the added PBS particles, which seems unrealistically high. An alternative explanation is that the activity of microbial degraders decreased over time, possibly reflecting decreases in the availability of nitrogen (N) or phosphorous (P) during long-term static incubations under laboratory conditions^69^. In contrast to plant or microbial biomass as substrates, PBS neither contains N nor P. Microbes utilizing PBS-derived breakdown products therefore need to acquire N and P from the surrounding soil. Possible N and P limitations and the slow release of carbon substrates from PBS could have caused microorganisms to primarily use PBS-derived carbon catabolically rather than anabolically, as build-up of many biomolecules and cell proliferation require sufficient supply not only of C but also N and P. Nutrient limitations can be tested for in future studies comparing biodegradation in soils with and without fertilization with N and P. Besides nutrient limitations, the activity of biodegrading microorganisms colonizing the PBS particles may have decreased also because of slow acidification of the PBS particle surfaces: hydrolysis of ester bonds at circumneutral pH releases of an equimolar number of protons. We propose that future studies should systematically assess these potential limitations on polymer biodegradation in soils by utilizing the analytical toolset developed herein.

Based on past mineralization studies of cellulose in soils, we ascribed high initial mineralization rates up to approximately 14 days of soil incubation to the microbial utilization of cellulose and the second phase with lower mineralization rates to turnover of cellulose ^13^C that was incorporated into microbial biomass. To support this interpretation, we also applied the above-described carbon flux model to the cellulose mineralization data, which predicted complete consumption of the cellulose after 16 days of incubation (i.e., less than 1% of the added cellulose remained) (Fig. 5d). The fitted CUE value of 0.37 was in good agreement with the amount of cellulose C_mineralized_ after 20 days, as well as previously published CUE of cellulose in soils^69,70^ (see Supplementary Note 7 for more detail). The cellulose mineralization data was well described with a single and constant rate constant for hydrolytic breakdown of cellulose. Since cellulose cannot be extracted from soils using our method, we could not experimentally confirm that C_polymer residual_ for cellulose was indeed negligible at the end of the incubations.

## Discussion

The herein-presented approach of using ^13^C-labelled PBS and cellulose allows to selectively track their carbon during biodegradation in soils. This tracking includes being able to continuously quantify polymer mineralization (i.e., C_mineralized_) during incubations in soils and, after terminating the incubations, to quantify the non-mineralized residual polymer-added carbon that remains in the soil, (i.e., C_non-mineralized_). As such, this approach allows closing the mass balance on polymer-added carbon over long-term soil incubations, which was impossible in previous work using non-labelled polymers. By using polymer variants which are synthesized to contain the ^13^C-label in the different monomer units that make up the polymer, it is further possible to unequivocally demonstrate that carbon from all monomeric building blocks is microbially utilized during polymer biodegradation, as shown herein for PBS. This monomer-specific ^13^C-labelling approach is particularly relevant for polymers containing monomeric units that may resist microbial utilization. While the availability and costs of using ^13^C-labelled polymers and the need for isotope-sensitive instrumentation may limit its broad applicability, we propose that the ^13^C-labelling approach be adopted in future studies on polymer biodegradation in soils to provide additional insights into the biodegradation process compared to those obtained with existing approaches using non-labelled polymers.

We showed how these stable carbon isotope-sensitive analyses can be combined with extraction and quantification of residual polymer in the soil, C_polymer residual_, which do not require the ^13^C label. The quantification of C_polymer residual_ may prove essential for the correct interpretation of the overall biodegradation data, as shown herein for PBS. We therefore propose that future studies assessing biodegradation of polymers extractable from soils ought to complement respirometric analyses with extracting and quantifying residual polymers in the soils at the end of the incubations. We validated the extraction method also for PBAT, another biodegradable polyester^71^, and expect the extraction method to be broadly applicable to biodegradable and conventional polymers that dissolve in chloroform. Quantification of C_polymer residual_ also allows indirect assessment of how much of the polymer carbon has been transferred into microbial biomass, C_biomass_. While C_biomass_ is determined only indirectly by subtracting C_polymer residual_ from C_non-mineralized_, we consider this estimate of C_biomass_ to be more robust than previous approaches that infer C_biomass_ from quantifying specific microbial biomolecules/biomarkers that are extracted from the soil^72–75^. Quantification of C_biomass_ using these latter approaches is challenging given that stabilities and extraction efficiencies of the selected biomolecules/biomarkers from soils are often unknown and that extracted amounts of these molecules are not readily convertible to total carbon in C_biomass_. We note that the traditional method to quantify biomass by soil fumigation with chloroform and subsequent extraction of biomass is especially ill-suited in this case, given that many biodegradable polyesters dissolve in chloroform and thus are expected to interfere with this measurement. Taken together, the presented approach overcomes analytical limitations encountered when studying biodegradation of non-labelled polymers in soils.

The use of ^13^C-labelled polymers in specific, mechanistic case studies on biodegradation, such as the one presented herein, also opens numerous possibilities to use additional stable carbon isotope-sensitive analytics to obtain additional insights into the biodegradation process. These include, but are not limited to, ^13^C-sensitive surface-analysis techniques (e.g., NanoSIMS and stable isotope Raman spectromicroscopy)^52,76–78^ which can potentially be used to image incorporation of polymer carbon into microbial cells on the polymer surface at the nm to µm scale, isotopic analysis of biomolecules and biomarkers (such as phospholipid fatty acids) extracted from the incubation medium^79–81^, and DNA-stable isotope probing^50,82,83^, as well as combinations thereof.

This work focused on PBS in the context of assessing biodegradation of agricultural films in soils. We demonstrated that PBS biodegraded extensively over the course of the incubation, in contrast to a recent report classifying PBS as non-biodegradable in soils^84^. However, the approach presented herein is also applicable to other synthetic, as well as bio-based, biodegradable polyesters used in agricultural products. The presented workflow will allow to systematically study polymer-specific properties that control rates and extents of biodegradation in soils. While experiments presented in this study used a single soil for method development and validation, this work lays the analytical foundation for future work that systematically assesses variations in the biodegradation rates and extents of specific polymers between different soils. Identifying variations and linking them to soil properties, such as the type and abundance of specific microbial degraders, will advance a more complete understanding of the biodegradation of agricultural films in soils. We propose that the approach presented herein may also be used to identify technologies that falsely claim to render non-biodegradable polymers biodegradable without solid scientific evidence. These technologies include pro-oxidant additives for plastics composed of conventional polyolefins (i.e., PE and polypropylene).

Beyond PBS and other biodegradable polyesters in soils, we consider the presented analytical approach based on using polymer ^13^C-labelling to be universally applicable to studies of polymer biodegradation in other open environments, such as marine and freshwater sediments, as well as in engineered systems, including compost and wastewater treatment plants. Finally, the approach will not only lead to a more complete picture of biodegradation of existing polymers but also provide guidance to ongoing efforts to develop sustainable polymers with desired biodegradability characteristics tailored to specific receiving environments. Using biodegradable polymers in specific applications known to have a high probability of plastics entering the open environment is a critical component in overcoming environmental plastic pollution^32^.

## Methods

All data were analyzed using Excel (version 16.58) and R (version 4.1.1) via RStudio (version 1.4), and figures were prepared using Adobe Illustrator (version 23) and R via RStudio.

### Poly(butylene succinate), monomers, and cellulose

**Fig. 6:**
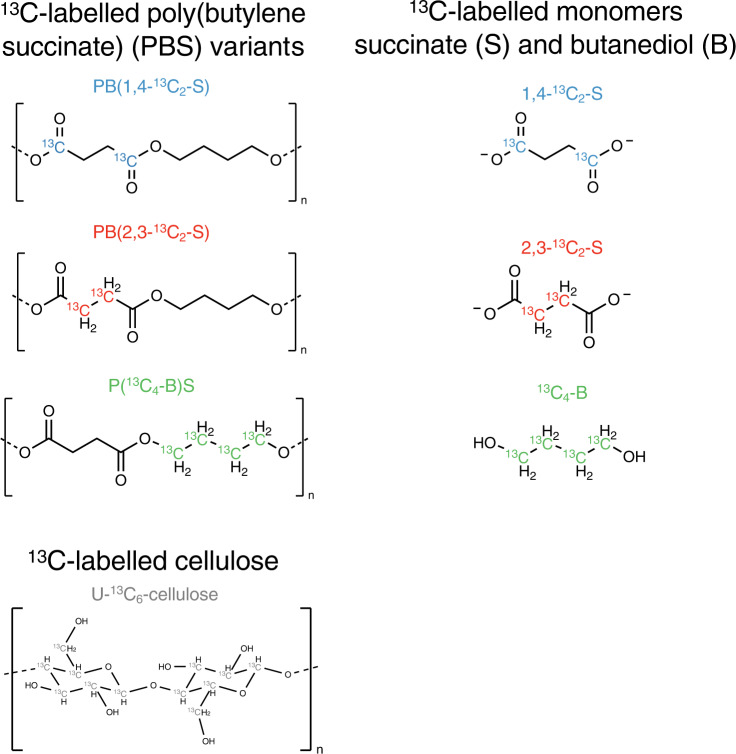
Chemical structures of the ^13^C-labelled poly(butylene succinate), corresponding monomers, and cellulose used in experiments. Substrates include the three variants of ^13^C-labelled poly(butylene succinate) (PBS) (i.e., PB(1,4-^13^C_2_-S); PB(2,3-^13^C_2_-S); and P(^13^C_4_-B)S), the corresponding ^13^C-labelled monomers of succinate (S) and butanediol (B) (i.e. 1,4-^13^C_2_-S; 2,3-^13^C_2_-S; and ^13^C_4_-B), and uniformly ^13^C-labelled cellulose.

Figure 6 shows the chemical structures of the three tested ^13^C-labelled PBS variants, their corresponding monomers (i.e., 1,4-^13^C_2_-S, 2,3-^13^C_2_-S and ^13^C_4_-B), and ^13^C-labelled cellulose.

The three position-specifically ^13^C-labelled PBS variants (i.e., PB(1,4-^13^C_2_-S), PB(2,3-^13^C_2_-S) and P(^13^C_4_-B)S) were synthesized by polycondensation^85^, using the respective ^13^C-labelled and non-labelled monomers –1,4-butanediol (B) and succinic acid (S)– in relative amounts to achieve the desired extent of ^13^C-labelling. Non-labelled B and S and the two variants of position-specifically ^13^C-labelled S (i.e., 1,4-^13^C_2_-S and 2,3-^13^C_2_-S), as well as uniformly ^13^C-labelled B (i.e., ^13^C_4_-B) (all of analytical grade and with ≥ 99% ^13^C-labelling extents at the indicated positions) were obtained from Sigma Aldrich and used as received. The synthesized PBS variants had an overall ^13^C atom percent (%^13^C) of about 3.5 atom% (see Table 1) as defined as:

3 $$\%{\,\!}^{13}{{{{{\rm{C}}}}}}=\frac{({\,\!}^{13}{{{{{\rm{C}}}}}}/{\,\!}^{12}{{{{{\rm{C}}}}}})}{1\,+({\,\!}^{13}{{{{{\rm{C}}}}}}/{\,\!}^{12}{{{{{\rm{C}}}}}})}$$

where ^13^C/^12^C is the molar ratio of the carbon isotopes ^13^C and ^12^C in the PBS. This ratio can also be expressed as a carbon isotopic signature (δ^13^C (‰)) when related to the ^13^C/^12^C ratio of a standard material, Vienna Pee Dee Belemnite (VPDB), with an ^13^C/^12^C ratio of 0.0112372:

4 $${{{{{{\rm{\delta }}}}}}}{\,\!}^{13}{{{{{\rm{C}}}}}}=\left(\frac{({\,\!}^{13}{{{{{\rm{C}}}}}}/{\,\!}^{12}{{\rm{C}}})_{{{{{{\rm{sample}}}}}}}}{({\,\!}^{13}{{{{{\rm{C}}}}}}/{\,\!}^{12}{{{{{\rm{C}}}}}})_{{{{{{\rm{VPDB}}}}}}}}-1\right) \cdot 1000$$

Given that the extent of ^13^C-labelling of the tested PBS variants was only 3.5% (i.e., most carbons were not labelled) and that carbon kinetic isotope effects are small (i.e., in the range of 1.01–1.07^86^, and typically involving slower reaction of the heavy than lighter isotopologue), we considered ^13^C labelling to have a negligible effect on biodegradation rates on bulk PBS. Table 1 provides the carbon-isotopic compositions and key physicochemical properties of the three labelled PBS variants. Experiments were run with small PBS particles. To this end, synthesized PBS was cryo-milled and subsequently sieved to obtain the 100–300 μm size fraction for the incubations.

**Table 1:** Carbon-isotopic composition and key physicochemical properties of the three ^13^C-labelled poly(butylene succinate) (PBS) variants used in soil incubations

PBS variant	δ^13^C ^a^	%^13^C ^a^	M_n_; M_w_^b^	T_g_; T_m_^c^
	(‰)	(atom %)	(g mol^−1^)	(°C)
PB(1,4-^13^C_2_-S)	2219	3.49	18670; 55620	−35; 113
PB(2,3-^13^C_2_-S)	2191	3.46	17430; 52630	−35; 114
P(^13^C_4_-B)S	2212	3.48	18870; 56520	−34; 114

^a^Carbon isotopic signatures (δ^13^C) were determined by elemental analysis coupled to isotope-ratio mass spectrometry (EA-IRMS) and referenced to the ^13^C/^12^C ratio of Vienna Pee Dee Belemnite (VPDB; ^13^C/^12^C = 0.0112372). These isotopic signatures served to calculate the corresponding atom % of ^13^C according to Eqs. 3 and 4, respectively.

^b^Number averaged molecular weights (M_n_) and weight averaged molecular weights (M_w_) were determined by gel permeation chromatography.

^c^Glass transition temperatures (T_g_) and melting temperature (T_m_) were determined by differential scanning calorimetry.

Non-labelled cellulose was obtained from Sigma Aldrich, and uniformly labelled U-^13^C_6_-cellulose (^13^C-labelled to an extent of ≥ 97%) was purchased from IsoLife (Netherlands). Labelled and non-labelled cellulose were mixed in a 1:9 mass ratio and ball-milled with zirconia beads to obtain a fine cellulose powder that was added to the soil for incubations. Dilution with non-labelled cellulose served to allow adding the same amounts of cellulose and PBS to the respective soil incubations while circumventing overly high δ^13^C values of CO_2_ that would have resulted from adding only fully labelled cellulose.

### Soil

An agricultural soil from the Agricultural Center Limburgerhof (Germany) was used for the incubation experiments. The soil was classified as a sandy clay loam based on the USDA soil texture classification, with a particle size distribution of 54.9 mass% sand (50 μm–2 mm), 12.3 mass% silt (2–50 μm), and 30.8 mass% clay (<2 μm). The organic carbon and total nitrogen contents were 1.14% and 0.11% by weight, respectively. The δ^13^C value of the soil carbon was −26.1 (±0.6) ‰, as determined by EA-IRMS of soil aliquots. After collection, the soil was air-dried, 2 mm sieved, and subsequently stored at 4 °C prior to use in the incubations. For the incubations, the water content of the soil was adjusted to 45% (by mass) of the maximum water holding capacity (WHC_max_ = 37.3 g_water_ 100 g_dry soil_^−1^) with MilliQ water (resistivity of ≥18.2 MΩ cm).

### Analytical workflow

**Fig. 7:**
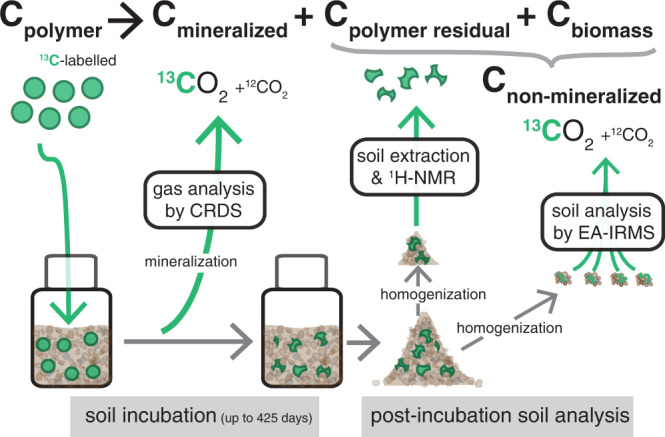
Analytical workflow to study biodegradation of ^13^C-labelled poly-(butylene succinate) (PBS) in soils. Following addition of ^13^C-labelled PBS to soils, mineralization of PBS-added ^13^C to ^13^CO_2_ was continuously followed by analysis of the effluent gas from incubation bottles using isotope-specific cavity ring-down spectroscopy (CRDS). Following incubations, the total amount of non-mineralized PBS-added ^13^C that remained in the soil, C_non-mineralized_, was determined in chloroform-sonication treated soils by elemental analysis coupled to isotope ratio mass spectrometry (EA-IRMS). Finally, the amount of residual PBS, C_polymer residual_, was quantified by extracting remaining PBS from soils in chloroform-methanol, followed by quantification of the extracted PBS in deuterated chloroform using quantitative proton nuclear magnetic resonance spectroscopy (^1^H NMR). We note that the depiction of spherical PBS particles with uniform size added to the soil was chosen for simplicity and does not reflect the actual size distribution and likely various shapes of actually added PBS particles.

The analytical workflow consisted of three parts: (i) an incubation system that allowed for continuous quantification of the mineralization rates of PBS, its monomers or cellulose into ^13^CO_2_ (i.e., C_mineralized_ in Eq. 1), (ii) quantification of total PBS-derived ^13^C and cellulose-derived ^13^C that remained in soils at the end of the incubation (i.e., C_non-mineralized_ in Eq. 2) and (iii) quantification of residual PBS that remained in the soil (i.e., C_polymer residual_ in Eqs. 1 and 2). The analytical workflow is schematically depicted in Fig. 7.

### Quantifying PBS, monomer, and cellulose mineralization, C_mineralized_

We determined mineralization of ^13^C-labelled PBS variants, monomers, and cellulose (triplicate bottles for each substrate) in an automated soil incubation system^42,52^. We simultaneously ran triplicate control incubations containing only soil but no added substrate. The incubation system had a maximum capacity of 36 incubation bottles (250 mL glass Schott bottles) which were housed in a temperature-controlled incubator at 25.0 ± 0.2 °C in the absence of light. The system was operated in continuous flow-through mode in which humidified, well-mixed ambient air was continuously pulled through the incubation bottles at a controlled flow rate (24 mL min^−1^) (i.e., flushing mode). We used an array of three-way solenoid valves (type 6122, Burkert) controlled by an automated logic controller (Arduino) to divert the flow from one incubation bottle at a time to the inlet of an isotope-sensitive cavity ring-down spectroscopy analyzer (CRDS; model G2201*i*, Picarro) for quantification of ^13^CO_2_ and ^12^CO_2_. The flow rate for the analysis in the CRDS was identical to the flushing flow rate (i.e., 24 mL min^−1^) to ensure that mineralization rates in all bottles were in steady-state equilibrium with the volumetric flow rate of the CRDS at any given time during the incubation. This setup reduced the analysis time because ^13^CO_2_ formation rates in a given incubation bottle could be immediately determined after it was connected to the CRDS (i.e., there was no accumulation of CO_2_ in the bottles between measurement timepoints and hence no long flushing was required prior to gas analysis). Mineralization of PBS and cellulose was initially measured at a higher frequency to capture initial biodegradation at high temporal resolution. At later stages of the incubation when mineralization had slowed down, we only periodically quantified mineralization rates. In between these analyses, we detached the incubation bottles from the analysis system, covered the bottles with aluminum foil, and stored them at 25 °C in the dark. When we re-attached the bottles to the system, we allowed for the soils to re-attain steady state equilibrium in their mineralization rates with the flow conditions through the incubation vials for at least two days before quantifying ^13^CO_2_ and ^12^CO_2_ concentrations in the incubation efflux gas. We periodically determined the water contents of the soils in these long-term PBS and cellulose incubations gravimetrically, and, in case of evaporative losses, we re-adjusted the water contents to initial values by adding MilliQ water onto the soil surface with a 50 mL spray bottle. As a result, there were only small fluctuations in the soil water contents over the course of the incubations (i.e., water contents decreased at most by 3 percentage points from 45 to 42% of the maximum water holding capacity). The measurement system is described in more detail in the Supplementary Note 8.

Each experiment was initiated by adding moisture-adjusted soil (equivalent to 100 g dry weight) to each incubation bottle and by pre-incubating these bottles connected to the flow-through system for at least six days before adding the ^13^C-labelled substrates. This pre-incubation period allowed the soils to reach a constant basal respiration rate and thereby minimized potential interference from elevated SOM mineralization following soil handling on the initial substrate mineralization rates. Following addition of one of the ^13^C-labelled PBS variants, the monomers, or cellulose to the soil (see below for details), we quantified the ^13^CO_2_ and ^12^CO_2_ concentrations in the effluent gas from each incubation bottle, switching between different incubation bottles every ten minutes. For each 10-min measurement period, we averaged the ^13^CO_2_ and ^12^CO_2_ concentrations over the last three minutes of that period into single ^13^CO_2_ and ^12^CO_2_ concentrations at that time point. Mineralization of each substrate was tested in triplicate soil incubations and corrected for background SOM mineralization measured in triplicate control bottles containing soils but no added substrate. Details on the calculations are given below in a separate subsection.

One of the three position-specifically ^13^C-labelled PBS variants (100 mg per incubation bottle) were directly mixed as fine particles into the pre-incubated soils (total of 100 g dry weight per incubation bottle) to obtain a homogenous PBS distribution in the soil. Triplicate incubations were run for each of the three PBS variants up to 319 days, when we removed one bottle for each variant and analyzed the soil for remaining ^13^C added as PBS (as detailed below). Mineralization in the remaining two bottles for each PBS variant was followed for a total of 425 days of incubation.

We dissolved each of the ^13^C-labelled monomers (i.e., 1,4-^13^C_2_-S, 2,3-^13^C_2_-S; and ^13^C_4_-B) in MilliQ water to concentrations of ~4.25 mM and adjusted the pH of the solutions to 7.0 ± 0.3 using 0.1 M NaOH. This pH adjustment prevented addition of acidic solutions to the soils which could have resulted in undesired dissolution of soil carbonates and subsequent release of CO_2_ that would not have been accounted for by control incubations. We subsequently added 1 mL of each monomer solution (corresponding to 4.0 μg butanediol or 5.0 μg succinate per g dry weight soil) to the soil (total of 100 g dry weight per incubation bottle) through the gas-tight septa in the lids of the incubation bottles using stainless steel needles and a glass syringe. During the addition, the bottles remained connected to the incubation system to avoid any loss of CO_2_ formed by rapid mineralization of the added monomers and to ensure that no ambient CO_2_ entered the bottles and interfered with measurement of the monomer mineralization. We followed monomer mineralization over a total of up to 14 days.

Similar to PBS, we homogenously mixed the cellulose powder (addition of 100 mg total cellulose (i.e., 10 mg uniformly ^13^C-labelled and 90 mg non-labelled cellulose) per incubation bottle) into the soils (total of 100 g dry weight per incubation bottle). We followed mineralization in triplicates up to 139 days of incubation, when we removed one of the three incubation bottles from the system to quantify ^13^C added as cellulose that remained in the soil at that time (as detailed below). Cellulose mineralization in the remaining two bottles was followed up to a total of 254 days.

We calculated the mineralized amounts of PBS-, cellulose-, and monomer-^13^C during soil incubation as follows. In a first step, we determined the fractional contributions of carbon from the added ^13^C-labelled PBS, cellulose, or monomers (referred to as substrates) to the overall measured CO_2_ concentrations, *f*_substrate_:^50,87^

5 $${f}_{{{{{{\rm{substrate}}}}}}}=\frac{({{{{{{\rm{\delta }}}}}}}^{13}{{{{{{\rm{C}}}}}}}_{{{{{{\rm{soil}}}}}}+{{{{{\rm{substrate}}}}}}}-{{{{{{\rm{\delta }}}}}}}^{13}{{{{{{\rm{C}}}}}}}_{{{{{{\rm{soil}}}}}}})}{({{{{{{\rm{\delta }}}}}}}^{13}{{{{{{\rm{C}}}}}}}_{{{{{{\rm{substrate}}}}}}}-{{{{{{\rm{\delta }}}}}}}^{13}{{{{{{\rm{C}}}}}}}_{{{{{{\rm{soil}}}}}}})}$$

where δ^13^C_soil+substrate_, δ^13^C_soil_, and δ^13^C_substrate_ represent the carbon isotopic signatures of CO_2_ formed in incubations containing soil with added substrate (subscript soil+substrate), of CO_2_ from control incubations without added substrate (subscript soil), and of the added ^13^C-labelled substrate (subscript substrate), respectively. The isotopic signatures were expressed as δ^13^C values referenced to VPDB for PBS and cellulose incubations (see Table 1 for PBS values), and as ^13^C atom% for monomer incubations (due to their higher extents of ^13^C-labelling).

In a second step, we used these fractions to calculate the amounts of PBS-, monomer- and cellulose-derived CO_2_, [CO_2_]_substrate_ (μmol CO_2_ mol^−1^ air), from the total CO_2_ measured during sample incubations, [CO_2_]_soil+substrate_ (μmol CO_2_ mol^−1^ air):

6 $${[{{{{{{\rm{CO}}}}}}}_{2}]}_{{{{{{\rm{substrate}}}}}}}={f}_{{{{{{\rm{substrate}}}}}}} \cdot {[{{{{{{\rm{CO}}}}}}}_{2}]}_{{{{{{\rm{soil}}}}}}+{{{{{\rm{substrate}}}}}}}$$

We converted these amounts to PBS-, cellulose-, or monomer-derived ^13^CO_2_, [^13^CO_2_]_substrate_ (μmol ^13^CO_2_ mol^−1^ air) based on the known ^13^C atom% of the respective substrate (Table 1 for PBS, text above for cellulose and monomers):

7 $$[{\,\!}{^{13}{{{{{\rm{CO}}}}}}_{2}}]_{{{{{{\rm{substrate}}}}}}}=[{{{{{{\rm{CO}}}}}}}_{2}]_{{{{{{\rm{substrate}}}}}}} \cdot {\%}^{13}{{{{{{\rm{C}}}}}}}_{{{{{{\rm{substrate}}}}}}}$$

In a third and final step, we converted [^13^CO_2_]_substrate_ to substrate−^13^C mineralization rates, *r*(^13^C_mineralized_) (μg ^13^C h^-1^):

8 $$r({\,\!}^{13}{{{{{{\rm{C}}}}}}}_{{{{{{\rm{mineralized}}}}}}})={[{\,\!}^{13}{{{{{\rm{CO}}}}}}_{2}]}_{{{{{{\rm{substrate}}}}}}} \cdot \frac{Q \cdot M}{V}$$

where Q (=1.44 L h^-1^) is the volumetric gas flow rate into the CRDS (and identical to the flow rate during flushing of incubation bottles), M (=13.003 g mol^−1^) is the molar mass of ^13^C, and V (=24.465 L mol^-1^) is the molar volume of air at 25 °C and 1 atm. Integrating the ^13^C-mineralization rates over the incubation time, *t* (hours), yielded cumulative amounts of substrate-^13^C mineralized, *n*(^13^C_mineralized_) (μg ^13^C), which were then normalized by the amounts of added substrate-^13^C, *n*(^13^C_added_) (μg ^13^C), to yield the percent of added substrate-^13^C that was mineralized, ^13^C_mineralized_ (%):

9 $${\,\!}^{13}{{{{{\rm{C}}}}}}_{{{{{{\rm{mineralized}}}}}}}=\frac{{\int }_{0}^{t}r({\,\!}^{13}{{{{{{\rm{C}}}}}}}_{{{{{{\rm{mineralized}}}}}}})\,{dt}}{n({\,\!}^{13}{{{{{{\rm{C}}}}}}}_{{{{{{\rm{added}}}}}}})} \cdot 100=\frac{n({\,\!}^{13}{{{{{{\rm{C}}}}}}}_{{{{{{\rm{mineralized}}}}}}})}{n({\,\!}^{13}{{{{{{\rm{C}}}}}}}_{{{{{{\rm{added}}}}}}})} \cdot 100$$

### Quantifying total PBS and cellulose-added ^13^C remaining in soil, ^13^C_non-mineralized_

Immediately after terminating the PBS and cellulose incubation experiments, we froze the soils in the incubation bottles (including the control soils containing no added substrates) at −80 °C, followed by freeze-drying the soils at 0.1 mbar for at least 36 hours. Each dried soil was then passed over a 2 mm sieve, followed by milling approximately half of each soil in a vibratory disk mill (model RS1, Retsch).

We treated the milled soils from the PBS incubations using chloroform-sonication to dissolve any residual PBS particles and thereby to homogenously re-distribute residual PBS in the soil (see Results section for details). First, we transferred milled soil subsamples of 5 g into 10 mL glass scintillation vials. We added chloroform to the vials to cover the soil subsamples and pulse-sonicated these samples on ice with an ultrasonic processor (Sonics) using a tapered microtip probe (1 mm tip diameter) for a total sonication time of 5 min (on time: 0.8 s / off time: 0.3 s, 500 W output, 40 % max. amplitude). The soil subsamples were subsequently re-dried by opening them in a ventilated hood overnight and then by freeze-drying at 0.1 mbar for a further 24 h. Finally, we weighed about 10 mg of each soil sample into tin capsules for EA-IRMS. Samples from control soils (i.e., soils without any added substrates) were treated in the identical manner to the corresponding soil with added substrates.

For soils from cellulose incubations, we directly weighed 10 mg of the milled soil into tin capsules for elemental analysis of soils linked to isotope ratio mass spectrometry (EA-IRMS).

The carbon isotopic signature (δ^13^C value referenced to VPDB) and carbon content (%C by mass) of each soil sample was determined with an elemental analyzer (Thermo Fisher FlashEA 1112) coupled to a continuous flow interface (Thermo Fisher ConFlo IV) and isotope-ratio mass spectrometer (Thermo Fisher Delta V Plus) (EA-IRMS). IRMS data were collected using Isodat (version 3.0). Helium was used as the carrier gas throughout the system at a flow rate of 80 mL min^−1^. Crimped tin capsules containing the soil samples were placed on a MAS 200 autosampler and successively introduced to the oxidative column of the EA (containing chromium oxide and silvered cobaltous oxide; operating temperature of 1020 °C). Following sample introduction, O_2_ gas was injected into the oxidative column for 3 s at a flow rate of 175 mL min^−1^ for combustion. The resulting gas was then carried through the reductive column (containing elemental copper; operating temperature = 650 °C), followed by a drying column packed with magnesium perchlorate. A GC column (length 3 m, packed with Porapak QS 50/80 mesh; operating temperature = 45 °C) served to separate CO_2_ from interfering gases prior to introduction to the IRMS.

The EA-IRMS was calibrated with primary standards NBS 22 (oil, δ^13^C = −30.03‰), IAEA-CH-6 (sucrose, δ^13^C = −10.45‰), and IAEA-CH-7 (polyethylene, δ^13^C = −32.15‰). The samples were run against CO_2_ reference gas with a δ^13^C = −28.23‰. For each sample run, we established linearity in the measured δ^13^C values by measuring various organic compounds with a range of isotopic signatures (nicotinamide (Thermo) with δ^13^C = −31.2‰; peptone (Sigma) with δ^13^C = −15.6‰; glucose (custom mixture, see below) with δ^13^C = 61.5‰). We prepared this glucose standard by mixing non-labelled glucose (Sigma) with ^13^C_6_-glucose (labelling extent 24–25%; Cambridge Isotope Labs). The final isotopic signature of the mixture was determined using EA-IRMS analysis and was found to be consistent between replicate subsamples of the glucose mixture (δ^13^C = 61.5 ± 0.3‰; 4 replicates). The carbon content was calculated using a calibration curve determined by measuring different amounts of a soil standard with known organic carbon composition (Bodenstandard Nr. 3, HEKAtech; %C_org_ = 4.401%).

We determined ^13^C_non-mineralized_ for PBS and cellulose from the EA-IRMS data as follows. First, the fractional contributions of the PBS- or cellulose-derived ^13^C to the total amount of carbon in the soil samples, *f*_polymer_, were determined. To this end, we used Eq. 5 (replacing subscript substrate with polymer as no monomers were measured) where δ^13^C_soil+polymer_, δ^13^C_soil_, and δ^13^C_polymer_ represented the isotopic signatures of CO_2_ formed during EA-IRMS analyses of soils containing added polymer (subscript soil+polymer), from soils without added polymer (subscript soil), and of the added ^13^C-labelled polymer (subscript polymer), respectively. All isotopic signatures here were expressed as δ^13^C values referenced to VPDB. In a second step, we calculated the absolute amounts of non-mineralized PBS- or cellulose-C, *n*(C_non-mineralized_) (μg C), based on *f*_polymer_, the total C of a given soil subsample quantified by EA-IRMS, *n*(C_soil+polymer_) (μg C), and the ratio in the masses of soil incubated, *m*(soil_incubated_) (g soil) and of soil used for EA-IRMS quantification, *m*(soil_EA-IRMS_) (g soil):

10 $$n\left({{{{{{\rm{C}}}}}}}_{{{{{{\rm{non}}}}}}-{{{{{\rm{mineralized}}}}}}}\right)={f}_{{{{{{\rm{polymer}}}}}}}{ \cdot n}({{{{{{\rm{C}}}}}}}_{{{{{{\rm{soil}}}}}}+{{{{{\rm{polymer}}}}}}}) \cdot \frac{m({{{{{{\rm{soil}}}}}}}_{{{{{{\rm{incubated}}}}}}})}{m({{{{{{\rm{soil}}}}}}}_{{{{{{\rm{EA}}}}}}-{{{{{\rm{IRMS}}}}}}})}$$

In a third step, we converted *n*(C_non-mineralized_) to the respective amounts of non-mineralized polymer−^13^C, *n*(^13^C_non-mineralized_) (μg ^13^C) (akin to Eq. 7):

11 $$n({\,\!}^{13}{{{{{{\rm{C}}}}}}}_{{{{{{\rm{non}}}}}}-{{{{{\rm{mineralized}}}}}}})=n\left({{{{{{\rm{C}}}}}}}_{{{{{{\rm{non}}}}}}-{{{{{\rm{mineralized}}}}}}}\right) \cdot {\%}^{13}{{{{{{\rm{C}}}}}}}_{{{{{{\rm{polymer}}}}}}}$$

Finally, *n*(^13^C_non-mineralized_) was normalized by the amounts of added polymer-^13^C, *n*(^13^C_added_) (μg ^13^C), to yield ^13^C_non-mineralized_ in % of the added polymer-^13^C:

12 $${\,\!}^{13}{{{{{\rm{C}}}}}}_{{{{{{\rm{non}}}}}}-{{{{{\rm{mineralized}}}}}}}=\frac{n({\,\!}^{13}{{{{{{\rm{C}}}}}}}_{{{{{{\rm{non}}}}}}-{{{{{\rm{mineralized}}}}}}})}{n({\,\!}^{13}{{{{{{\rm{C}}}}}}}_{{{{{{\rm{added}}}}}}})} \cdot 100$$

### Closing mass balances on polymer-^13^C over the course of soil incubations

We determined the total mass balance on PBS- and cellulose-^13^C, ^13^C_mass balance_ (%), over the course of the incubation by adding the mineralized and non-mineralized PBS- and cellulose-^13^C, ^13^C_mineralized_ and ^13^C_non-mineralized_:

13 $${\,\!}^{13}{{{{{\rm{C}}}}}}_{{{{{{\rm{mass}}}}}}\; {{{{{\rm{balance}}}}}}}={\,\!}^{13}{{{{{\rm{C}}}}}}_{{{{{{\rm{mineralized}}}}}}}+{\,\!}^{13}{{{{{\rm{C}}}}}}_{{{{{{\rm{non}}}}}}-{{{{{\rm{mineralized}}}}}}}$$

### Quantification of PBS remaining in soil, C_polymer remaining_

We quantified residual PBS in the soils, C_polymer residual_, at the end of the incubations as follows. In a first step, we extracted residual PBS from the soil. To this end, we transferred 2.5 g subsamples of the sieved and freeze-dried (but not milled) soils from the PBS incubation experiments on top of a layer of glass wool inside micro-Soxhlet extractors (8 mL volume). We subsequently extracted these soil subsamples for 8 h under reflux using a 90/10 vol% chloroform/methanol mixture. These conditions were previously shown to fully extract PBAT from soils^71^. We then completely removed the solvent from the extract under a stream of air and subsequently re-constituted the dried extracts in 1 mL deuterated chloroform. Prior to analysis of the reconstituted extracts, we added a known amount of 1,4-dimethoxybenzene (DMB) dissolved in deuterated chloroform to the extract as internal quantification standard.

In a second step, we quantified the extracted PBS by ^1^H NMR spectroscopy. NMR spectra were collected using TopSpin (version 3.4) and analyzed using MestReNova (version 14.2.3). All ^1^H NMR analyses were performed on a Bruker Avance III 400 MHz with 5 mm BBFO Z-Gradient probe. We used the following values for key acquisition parameters: P1 (applied pulse length) = 14 μs, NS (number of acquisition scans) = 128, DS (number of dummy scans) = 16, D1 (delay time between scans) = 15 s. To quantify the extracted amount of PBS in deuterated chloroform we calculated the molar ratio of PBS to DMB in each sample, *X*_PBS:DMB_:

14 $${X}_{{{{{{\rm{PBS}}}}}}:{{{{{\rm{DMB}}}}}}}=\frac{\sum {A}_{{{{{{\rm{PBS}}}}}}}}{\sum {A}_{{{{{{\rm{DMB}}}}}}}} \cdot \frac{n({\,\!}^{1}{{{{{{\rm{H}}}}}}}_{{{{{{\rm{DMB}}}}}}})}{n({\,\!}^{1}{{{{{{\rm{H}}}}}}}_{{{{{{\rm{PBS}}}}}}})}$$

where Σ*A*_PBS_ and Σ*A*_DMB_ correspond to the areas of PBS and DMB peaks in the ^1^H NMR spectrum that we used for quantification, *n*(^1^H_PBS_) and *n*(^1^H_DMB_) are the numbers of the respective protons used for quantification per PBS repeat unit or molecule of DMB in the positions used for quantification. For PBS, we used the protons on the 2,3-carbons of S (chemical shift δ = 2.62 ppm) and 1,4-carbons of B (δ = 4.12 ppm) (*n*^1^H_PBS_ = 8). For DMB, we used the protons on both the methoxy-carbons (δ = 3.77 ppm) and the aryl-carbons (δ = 6.84 ppm) (*n*^1^H_DMB_ = 10). Selected ^1^H NMR spectra of PBS and DMB in both pure deuterated chloroform and soil extracts in deuterated chloroform as well as the assignment of peaks to protons in PBS and DMB are shown in the Supplementary Note 5. We then calculated the PBS mass in each sample, *n*(PBS) (mg PBS) by multiplying *X*_PBS:DMB_ by the amount of DMB added into each extract, *n*(DMB_added_) (mol DMB), and the molar mass of one repeat unit of PBS (-B-S-; C_8_H_12_O_4_), *M*_PBS_ (= 172.18 g mol^-1^):

15 $$n\left({{{{{\rm{PBS}}}}}}\right)={X}_{{{{{{\rm{PBS}}}}}}:{{{{{\rm{DMB}}}}}}}{ \cdot n}\left({{{{{{\rm{DMB}}}}}}}_{{{{{{\rm{added}}}}}}}\right) \cdot {M}_{{{{{{\rm{PBS}}}}}}}$$

Finally, we multiplied *n*(PBS) by the carbon content of PBS by mass (%C_PBS_ = 55.8) to obtain the mass of residual PBS-C, *n*(C_PBS residual_) (mg C):

16 $$n\left({{{{{{\rm{C}}}}}}}_{{{{{{\rm{PBS\; residual}}}}}}}\right)=n\left({{{{{\rm{PBS}}}}}}\right) \cdot \frac{\%{{{{{{\rm{C}}}}}}}_{{{{{{\rm{PBS}}}}}}}}{100}$$

The residual PBS remaining in the soils at the end of the incubations, C_PBS residual_, expressed in percent (%) of total PBS added was then given as:

17 $${{{{{{\rm{C}}}}}}}_{{{{{{\rm{PBS\; residual}}}}}}}=\frac{n({{{{{{\rm{C}}}}}}}_{{{{{{\rm{PBS\; residual}}}}}}})}{n({{{{{{\rm{C}}}}}}}_{{{{{{\rm{added}}}}}}})} \cdot \frac{m({{{{{{\rm{soil}}}}}}}_{{{{{{\rm{incubated}}}}}}})}{m({{{{{{\rm{soil}}}}}}}_{{{{{{\rm{extracted}}}}}}})} \cdot 100$$

where *n*(C_added_) (mg C) is the mass of PBS-C added to the soil at the onset of the incubations and *m*(soil_incubated_) and *m*(soil_extracted_) (each in g soil) are the masses of soils used in the incubations and that were extracted, respectively.

Further information on the quantification methods, including assessment of linearity of peak responses to PBS concentrations as well as limits of detection and quantification, are provided in the Supplementary Note 5.

### Kinetic modeling of biodegradation data

**Fig. 8:**
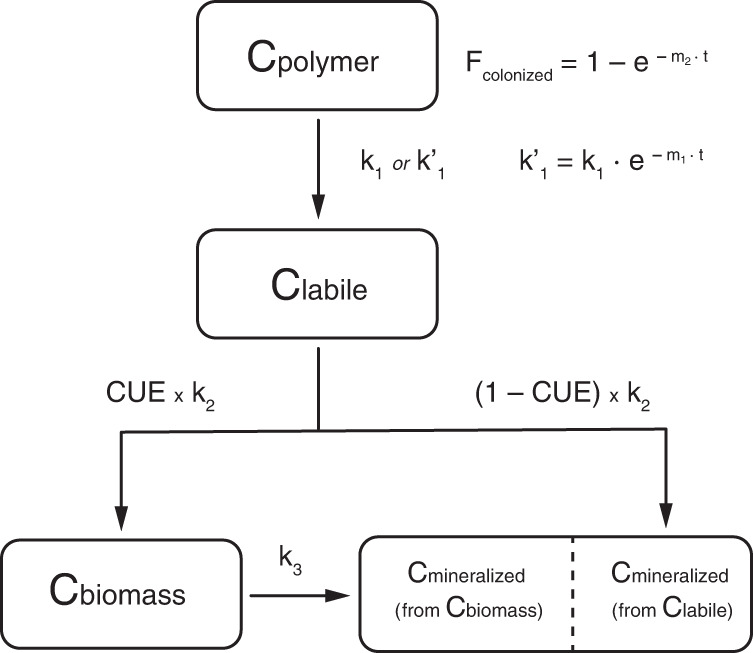
Carbon flux model used to describe poly(butylene succinate) (PBS) and cellulose biodegradation dynamics during soil incubation. Boxes represent carbon pools (C_*i*_) where C_polymer_ refers to carbon in bulk PBS and cellulose, C_labile_ is PBS- and cellulose-derived carbon in low molecular weight breakdown products, C_mineralized_ is PBS- and cellulose-derived carbon in CO_2_ (formed either directly catabolically or from the mineralization of microbial biomass that had first incorporated PBS- or cellulose-derived carbon), and C_biomass_ is carbon in the microbial biomass, microbial necromass and the soil organic matter pool. Arrows represent fluxes of carbon between pools and are labelled with the respective kinetic rate constants (*k*_*i*_); *k*_1_ and *k*_1_’ are the rate constants of depolymerization to form lower molecular weight compounds (C_labile_), *k*_2_ is the rate of microbial substrate utilization, and *k*_3_ refers to the mineralization rate of soil microbial biomass. F_colonized_ represents the fraction of the polymer surface which has been colonized by microbial degraders and which therefore is accessible for enzymatic breakdown. CUE is the carbon use efficiency and represents the fraction of total labile carbon taken up by microorganisms which is incorporated into microbial biomass. Rates of carbon transfer between pools are calculated based on differential equations, with kinetic parameters optimized by fitting experimental data of C_mineralized_, and additionally of C_polymer residual_ for some PBS model fits. The model is explained in more detail in the Supplementary Note 7.

To fit biodegradation data of PBS, the corresponding monomers, and cellulose, we developed a carbon flux model based on previously published models for soil organic matter degradation^88,89^, and our conceptual understanding of polyester biodegradation in soils^26^. Kinetic modeling was performed using COPASI (version 4.34). Namely, we included the following key steps of polyester biodegradation: (i) colonization of polyester surfaces by soil microorganisms, and the subsequent production and release of extracellular esterases leading to the hydrolytic depolymerization of PBS, (ii) the uptake and metabolic utilization of the low molecular weight PBS breakdown products by microorganisms for energy production under formation of CO_2_ and microbial biomass, and (iii) mineralization (turnover) of polyester-derived carbon previously incorporated into microbial biomass and biogenic soil organic matter (SOM). The carbon flux model is depicted schematically in Fig. 8.

More details, as well as full results, for the modeling are shown in the Supplementary Note 7. In brief, we set up the box model in the biochemical system simulation software COPASI^90^, and defined the rates of carbon flux between the carbon pools using ordinary differential equations. We then used experimental mineralization data from soil incubations of PBS, monomers, and cellulose, as well as extraction data for PBS, to fit several model variations to the experimental data. After importing the relevant experimental data, we performed the Parameter Estimation task in COPASI to determine the optimized values for the various model parameters. Using the optimized parameter values, we then performed forward simulations to model dynamics of the various carbon pools in each experiment using the COPASI task Time Course. The program files (type “.cps”) for the open source biochemical modeling program COPASI used herein are available from the ETH Zurich Research Collection at 10.3929/ethz-b-000544231.

Finally, for one PBS model, we conducted a sensitivity analysis of the model by varying the value of one fitted model parameter at a time, and subsequently determining the objective function value as a measure for the goodness-of-fit for the model (Supplementary Note 7).

### Reporting summary

Further information on research design is available in the Nature Research Reporting Summary linked to this article.

## Supplementary information

Supplementary Information

Reporting Summary

## Data Availability

The data presented in this manuscript and its supplementary information are available from the ETH Zurich Research Collection at 10.3929/ethz-b-000544231.
