# Supplementary material for: Biodegradation of poly(butylene succinate) in soil laboratory incubations assessed by stable carbon isotope labelling
Source: Nat Commun. 2022 Sep 28;13:5691. doi: 10.1038/s41467-022-33064-8 (PMC9519748; doi:10.1038/s41467-022-33064-8)
Supplement: Supplementary file 1 — Supplementary Information [file 41467_2022_33064_MOESM1_ESM.pdf]

Supplementary Information

Biodegradation of poly(butylene succinate) in soil laboratory incubations assessed by stable carbon isotope labelling

Taylor F. Nelson[1], Rebekka Baumgartner[1], Madalina Jaggi[2], Stefano M. Bernasconi[2], Glauco Battagliarin[3], Carsten Sinkel[3], Andreas Künkel[3], Hans-Peter E. Kohler[4], Kristopher McNeill[1], and Michael Sander[1*]

[1] Institute of Biogeochemistry and Pollutant Dynamics, ETH Zurich, 8092 Zurich, Switzerland

[2] Geological Institute, Department of Earth Sciences, ETH Zurich, 8092 Zurich, Switzerland

[3] BASF SE, Carl-Bosch-Strasse 38, Ludwigshafen 67056, Germany

[4] Environmental Microbiology, Swiss Federal Institute of Aquatic Science and Technology (Eawag), 8600 Dübendorf, Switzerland

*To whom correspondence should be addressed.

E-mail: michael.sander@env.ethz.ch

Phone: +41-(0)44 632 8314

Fax: +41-(0)44 633 1122

Supplementary Note 1

The three position-specifically ^{13}C-labelled PBS variants showed indistinguishable final cumulative ^{13}C mineralization extents (i.e., $C_{mineralized}$) after 425 days of soil incubation. We therefore concluded that microorganisms utilized carbon from the different positions in PBS to comparable extents. At the onsets of the soil incubations, however, the three PBS variants showed different mineralization rates, as discussed in detail in the Results section of the main text. We ascribed the differences in mineralization rates of the three PBS variants during the initial 7 days of incubation (i.e., initial mineralization rates decreased in the order PB(1,4-$^{13}C_2$-S) > P($^{13}C_4$-B)S > PB(2,3-$^{13}C_2$-S)) to differences in the microbial utilization of B and S monomers and low molecular weight BS oligomers that were present in the bulk PBS and that readily diffused out of the PBS into the soil. For a better visualization of these differences between the three PBS variants, we replotted the PBS mineralization rates and extents from Figure 1a and b in the main text in Supplementary Figure 1 on a double-logarithmic plot.

Following the initial maximum in mineralization rates during the first few days of incubation, the mineralization rates reached minima after about 7 days of incubation. From this point on, mineralization rates of all variants increased again, reaching a second maximum in mineralization rates at about 100 days of incubation. Supplementary Figure 1 shows that from 7 to about 40 days of incubation, ^{13}C mineralization rates differed among the three variants in the same order as observed during the first 7 days of soil incubation (i.e., PB(1,4-$^{13}C_2$-S) > P($^{13}C_4$-B)S > PB(2,3-$^{13}C_2$-S)). We ascribe the differences during this initial time period to the formation of microbial biomass in the soil with monomer- and position-specific extents to which ^{13}C was incorporated into microbial biomass, as discussed in more detail for the microbial utilization of the ^{13}C-labelled monomers in the soil (i.e., Figure 1c and d in the main text).

After about 40 days of incubation, mineralization rates were comparable between the three tested PBS variants. We interpret the apparent absence of variant-dependent mineralization rates during the later stages of incubation to reflect extensive conversion of PBS-derived ^{13}C to $^{13}CO_2$, irrespective of the position in PBS that carried the label. Extensive conversion to CO_2 is supported by the finding of only small $C_{biomass}$ at the end of the incubation. In addition, the modeling results suggest that biomass was formed only during the first 2-3 months of incubation. Extensive conversion to CO_2 beyond 100 days of incubation has two potential explanations. The first explanation is that the carbon use efficiencies (CUEs) were low and hence PBS-derived ^{13}C was primarily catabolic utilized and converted to $^{13}CO_2$. The second explanation is that the absence of observed position specificity in mineralization reflected that the rate at which PBS-derived ^{13}C cycled through the microbial biomass pool was larger than the rate at which PBS-derived carbon was provided to the microbial cells. In this second explanation, position- and monomer-specific utilization of carbon to form biomass may have continued beyond 100 days of incubation but was kinetically masked by the low supply rate of PBS-derived carbon to microbial cells.

As discussed in the main text, the causes for the decrease in PBS mineralization rates (and hence also the supply rate of PBS-derived carbon to microbial cells) beyond 100 days of soil incubation remain unclear. It is conceivable that the residual PBS was more difficult to enzymatically hydrolyze. It is also possible that conditions formed in the soils that started to limit the activity and possibly number of microbial degraders. For instance, limitations in N and P may have slowed down the formation of additional biomass and thus resulted in the preferential utilization of PBS carbon for energy production under formation of CO_2.

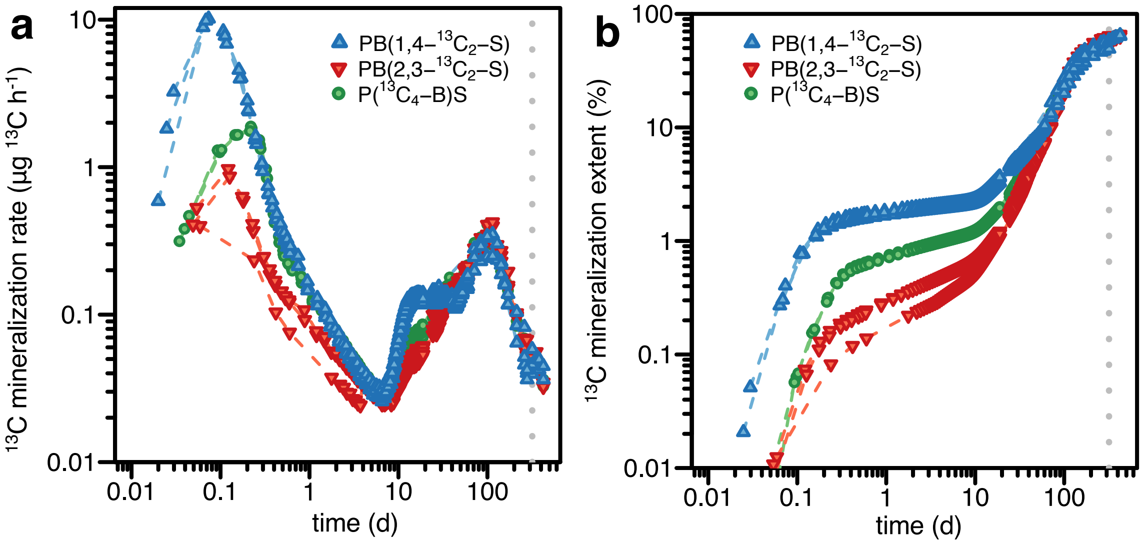

Supplementary Figure 1. Mineralization of ^{13}C-labelled poly(butylene succinate) (PBS) during soil incubations on a log-log scale. Mineralization rates (a) and cumulative extents (as % of added PBS-^{13}C) (b) of three position-specifically labelled variants of PBS (i.e., PB(1,4-$^{13}C_2$-S), blue up-triangles; PB(2,3-$^{13}C_2$-S), red down-triangles; and P($^{13}C_4$-B)S, green circles) during soil incubations. Dashed lines represent linear interpolations between measurement points of individual incubations. For each of the three PBS variants, incubations were run in triplicates up to 319 days (indicated by vertical grey dotted lines) and duplicates from 319 to 425 days. Data is replotted from Figure 1a, b in the main text, respectively, but plotted with both x and y axes converted to log-scales to highlight differences in the mineralization dynamics of the three PBS variants during the first two to three months of soil incubation.

Supplementary Note 2

The three different position-specifically ^{13}C-labelled PBS monomers (i.e., 1,4-$^{13}C_2$-S; 2,3-$^{13}C_2$-S; and $^{13}C_4$-B) showed distinct mineralization rates and extents during soil incubations, as described in the main text. Each of the three monomers reached a maximum in mineralization rate within the first few hours after monomer addition to the soil (Figure 1c, main text, inset). To facilitate comparisons of the mineralization dynamics of the three monomers, we have replotted the data from Figure 1c (main text) in Supplementary Figure 2 in which we normalized the measured mineralization rates of the monomers to the respective maximum mineralization rate of each of the monomers.

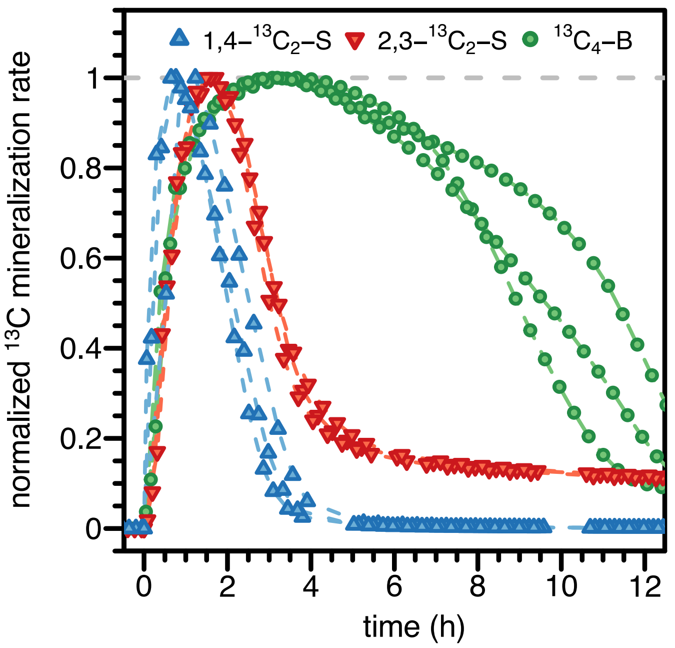

Supplementary Figure 2. Normalized mineralization rates of poly(butylene succinate) (PBS)-monomers. Mineralization rates of the two ^{13}C-labelled succinate monomers (i.e., 1,4-$^{13}C_2$-S, blue up-triangles; and 2,3-$^{13}C_2$-S, red down-triangles) and of the ^{13}C-labelled 1,4-butanediol ($^{13}C_4$-B, green circles), normalized to the maximum mineralization rates of the respective incubation bottle. Mineralization of the monomers was continuously followed in triplicate incubation bottles (for which measurements of each individual bottle are shown as a separate curve) for a total incubation time of 14 days. Note that this data is identical (except for the normalization) to the mineralization rate data shown in Figure 1c of the main text.

Supplementary Note 3

We rationalize the strong position-specificity of ^{13}C-mineralization rates of succinate with the metabolic flow of succinate carbons in the citric acid cycle (CAC) of aerobic soil microorganisms.[1] Following uptake and conversion of succinate to malate in the CAC, a fraction of the malate is decarboxylated first to pyruvate and subsequently to acetyl-CoA (see Supplementary Figure 3). In this process, both carboxylate groups in succinate are converted to CO_2. The acetyl-CoA is fed back into the CAC to convert oxaloacetate to citrate. The subsequent conversion of citrate to succinate involves the decarboxylation of both carboxylate groups in oxaloacetate, which also originate from the 1,4-carboxylate groups of succinate. According to this reaction pathway, two molecules of 2,3-$^{13}C_2$-succinate in the CAC cycle form

one molecule of succinate preferentially enriched in the ^{13}C-label and three molecules of $^{12}CO_2$ (plus one of $^{13}CO_2$) (Supplementary Figure 3a). Conversely, two molecules of 1,4-$^{13}C_2$-succinate are thus converted to one molecule of succinate preferentially reduced in the ^{13}C-label and three molecules of $^{13}CO_2$ (plus one of $^{12}CO_2$) (Supplementary Figure 3b).

These differences in the metabolic utilization of the carboxylate and methylene carbons in succinate can explain the extensive mineralization of ^{13}C in 1,4-$^{13}C_2$-S to $^{13}CO_2$, whereas much less $^{13}CO_2$ was formed from 2,3-$^{13}C_2$-S. The latter implies that the ^{13}C in 2,3-$^{13}C_2$-S was extensively incorporated into microbial biomass (i.e., formation of $C_{biomass}$, presumably by synthesis of fatty acids and lipids from acetyl-CoA and of carbohydrates from oxaloacetate; Supplementary Figure 3). We propose that the aerobic metabolic utilization of $^{13}C_4$-butanediol by soil microorganisms may have involved its oxidation to fully ^{13}C-labelled succinate, which subsequently entered the CAC.[2] This proposed pathway is supported by the slight delay in the maximum mineralization rates of $^{13}C_4$-B relative to 1,4- and 2,3-$^{13}C_2$-S (Supplementary Figure 2) as well as the intermediate rates and extents of mineralization of the uniformly labelled $^{13}C_4$-B as compared to 1,4-$^{13}C_2$- and 2,3-$^{13}C_2$-S (Figure 1c, d in the main text).

a)

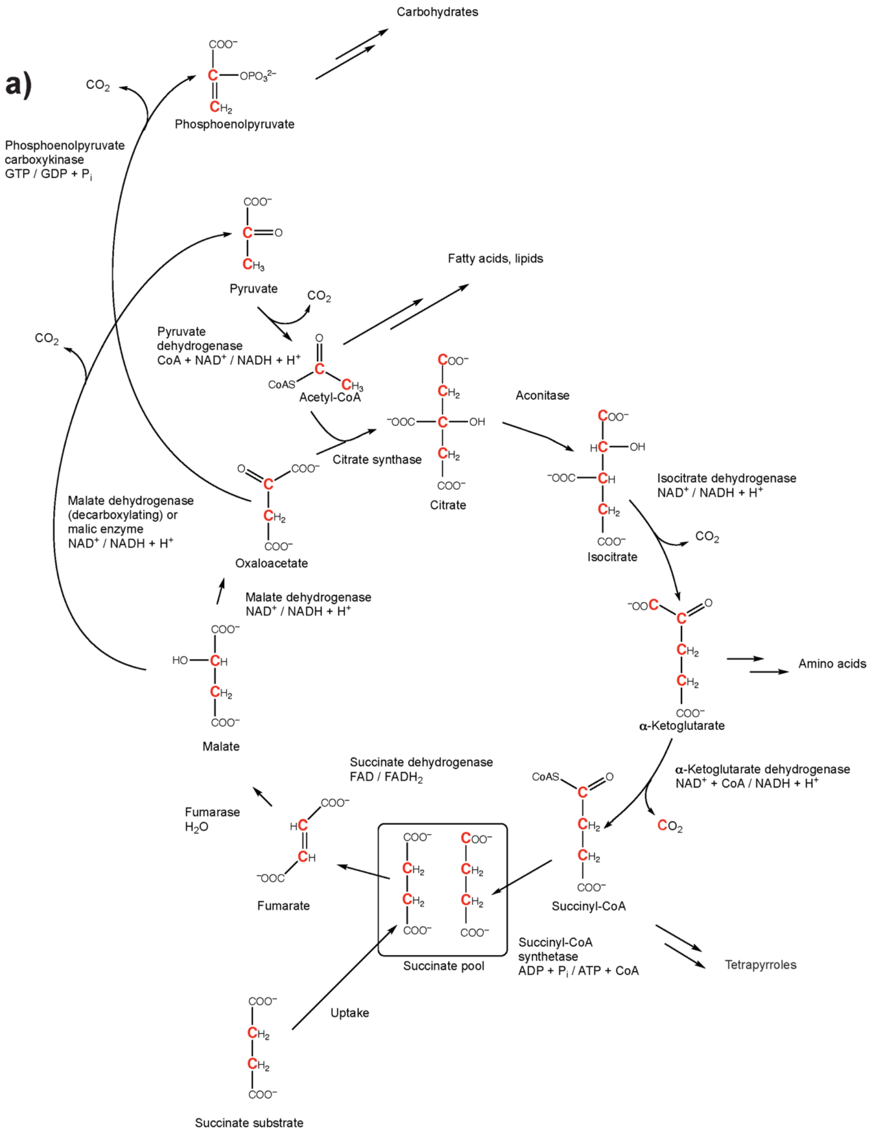

Carbohydrates
Phosphoenolpyruvate
Phosphoenolpyruvate carboxykinase
GTP / GDP + P_i
CO_2
Pyruvate
Pyruvate dehydrogenase
CoA + NAD^+ / NADH + H^+
Acetyl-CoA
Fatty acids, lipids
Citrate synthase
Citrate
Aconitase
Isocitrate
Isocitrate dehydrogenase
NAD^+ / NADH + H^+
α-Ketoglutarate
Amino acids
α-Ketoglutarate dehydrogenase
NAD^+ + CoA / NADH + H^+
Succinyl-CoA
Succinyl-CoA synthetase
ADP + P_i / ATP + CoA
Tetrapyrroles
Succinate pool
Uptake
Succinate substrate
Succinate dehydrogenase
FAD / $FADH_2$
Fumarate
Fumarase
H_2O
Malate
Malate dehydrogenase
NAD^+ / NADH + H^+
Oxaloacetate
Malate dehydrogenase (decarboxylating) or malic enzyme
NAD^+ / NADH + H^+

b)

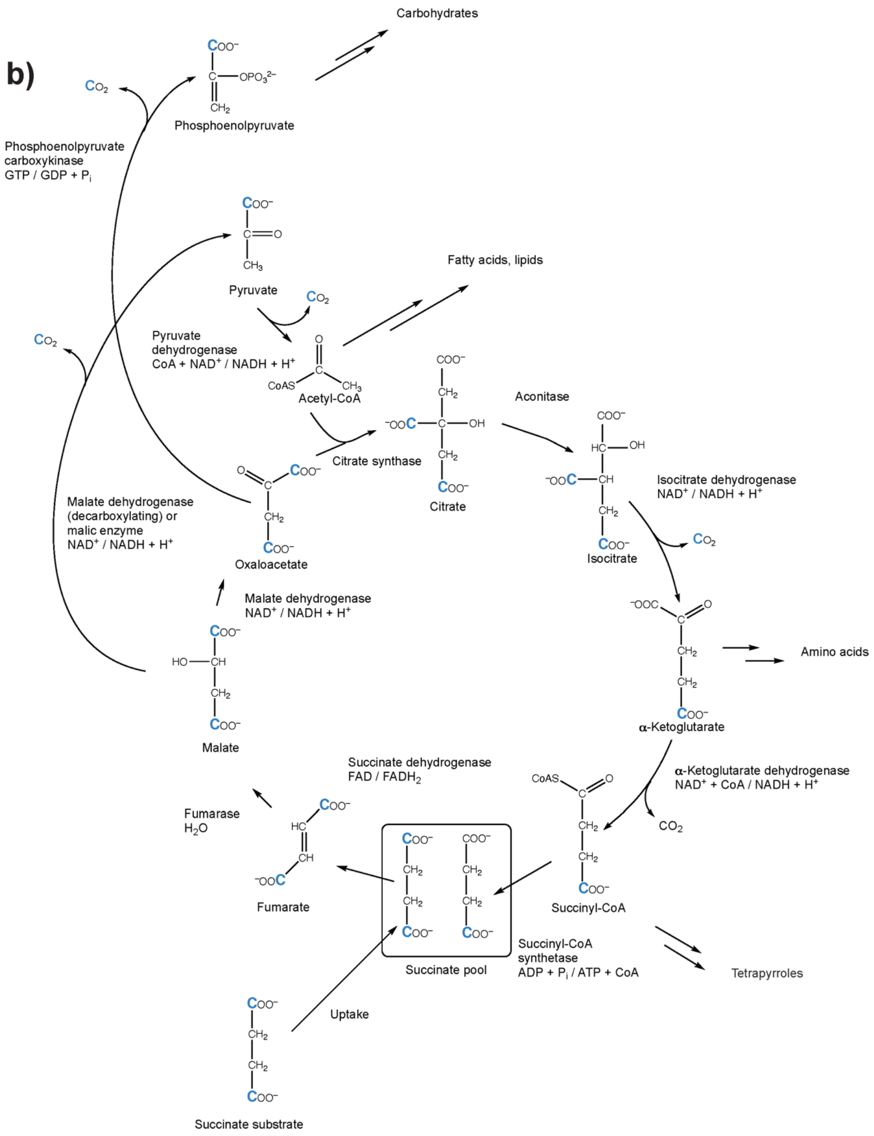

Carbohydrates
Phosphoenolpyruvate
Phosphoenolpyruvate carboxykinase
GTP / GDP + P_i
CO_2
Pyruvate
Pyruvate dehydrogenase
CoA + NAD^+ / NADH + H^+
Acetyl-CoA
Fatty acids, lipids
Citrate synthase
Citrate
Aconitase
Isocitrate
Isocitrate dehydrogenase
NAD^+ / NADH + H^+
α-Ketoglutarate
Amino acids
α-Ketoglutarate dehydrogenase
NAD^+ + CoA / NADH + H^+
Succinyl-CoA
Succinyl-CoA synthetase
ADP + P_i / ATP + CoA
Tetrapyrroles
Succinate pool
Uptake
Succinate substrate
Succinate dehydrogenase
FAD / $FADH_2$
Fumarate
Fumarase
H_2O
Malate
Malate dehydrogenase
NAD^+ / NADH + H^+
Oxaloacetate
Malate dehydrogenase (decarboxylating) or malic enzyme
NAD^+ / NADH + H^+

Supplementary Figure 3. Position-specific metabolic pathway of succinate carbons in the citric acid cycle. Depiction of the citric acid cycle, highlighting the differences in the metabolic utilization of carboxylate and methylene carbons of succinate.[1] Both panels a and b show the same overall cycle, with colored carbons highlighted to track the position-specific metabolic conversion of either the methylene carbons (i.e., 2- and 3- positions) (panel (a), red highlights) or carboxylate carbons (i.e., 1- and 4- positions) (panel (b), blue highlights) of succinate. The carboxylate carbons are preferentially converted to CO_2 while the methylene carbons preferentially remain in the citric acid cycle.

Supplementary Note 4

Method development for quantification of $^{13}C_{non\text{-}mineralized}$ in soil

We used soil samples to which we added known amounts of P($^{13}C_4$-B)S particles to develop and validate the chloroform-sonication treatment step to uniformly distribute of PBS-^{13}C in soils prior to subsampling the soil for EA-IRMS analyses. This treatment step and the resulting accurate and precise quantification of PBS-^{13}C has been discussed in the Results section (Figure 2) in the main text. We restricted our discussion in the main text to the P($^{13}C_4$-B)S recovery data for three soil samples with concentrations of 1, 3, or 10 mg P($^{13}C_4$-B)S per 10 g of soil. We compared recoveries from soil subsamples that were collected from a soil that was not chloroform-sonication-treated and from 5 g of soil after it was chloroform-sonication-treated. The subsamples collected for EA-IRMS analysis had a mass of 10 mg.

Here we present results of additional addition-recovery assessments for the soil sample with the intermediate concentration of 0.3 mg P($^{13}C_4$-B)S g^{-1} soil. In these additional assessments, we chloroform-sonication treated only 1 and 3 g soil samples (instead of 5 g) of the soil. As before, we analyzed five 10 mg aliquots of each of the chloroform-sonication treated soil subsamples using EA-IRMS.

Supplementary Figure 4 shows the recovery of the added P($^{13}C_4$-B)S for the five replicate aliquots taken from the 1, 3 and 5 g of soil that we treated by chloroform-sonication (note that the 5 g data is replotted from Figure 2 in the main text). As discussed in detail in the main text, we achieved complete recovery when chloroform-sonication treating a 5 g soil subsamples

(i.e., recovery of added P($^{13}C_4$-B)S of 97 ± 4% (mean ± standard deviation of five replicates)). We also achieved full recovery of added P($^{13}C_4$-B)S when treating only 3 g of soil by chloroform-sonication, followed by EA-IRMA analysis of five aliquots (i.e., recoveries of 101 ± 3% (mean ± standard deviation of five replicates)). However, when we chloroform-sonication treated only 1 g of soil that had a concentration of 0.3 mg P($^{13}C_4$-B)S g^{-1} soil, the recovery was incomplete (i.e., 89 ± 1% (mean ± standard deviation of five replicates)). While the recoveries were inaccurate, they were precise. This finding implies that taking and subsequently chloroform-sonication treating only 1 g subsamples from the total of 10 g of soil to which we had added 3 mg P($^{13}C_4$-B)S g^{-1} was too small to ensure that the amount of P($^{13}C_4$-B)S particles in the homogenized sample was representative of the complete soil. Based on this finding, we chose to chloroform-sonication treat 5 g soil subsamples from the PBS incubation experiments to accurately quantify $C_{non\text{-}mineralized}$ using EA-IRMS.

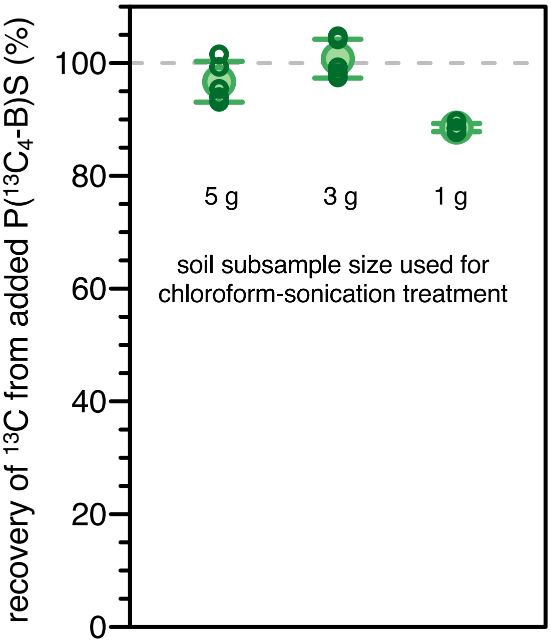

Supplementary Figure 4. Effect of soil subsample size used in chloroform-sonication treatment on recovery of poly(butylene succinate) (PBS)-^{13}C in soil. Recovery of ^{13}C from butanediol-^{13}C labelled PBS (i.e., P($^{13}C_4$-B)S) from a soil sample with a total amount of 10 g and a concentration of 0.3 mg P($^{13}C_4$-B)S g^{-1} soil (obtained by adding 3 mg P($^{13}C_4$-B)S to the 10 g of soil). The recovery was determined by EA-IRMS on five replicate aliquots (10 mg) that were collected from 5, 3, or 1 g chloroform-sonication treated soil subsamples of the 10 g soil. The individual recoveries for five replicates are plotted as open circles. The closed circles and

error bars are the means and the standard deviation of the five replicate samples. Data for the 5 g subsamples is replotted from Figure 2 in the main text.

Supplementary Note 5

Quantification of residual PBS in soils

We first prepared PBS standards in deuterated chloroform ranging in concentration from 0.12 to 5 mg PBS mL^{-1} to establish linearity in the ^1H NMR peak area responses to PBS concentrations. Supplementary Figure 5a shows the chemical structures of PBS and the internal quantification standard 1,4-dimethoxybenzene (DMB) as well as the protons of the two compounds that we selected for quantification. A representative NMR spectrum for a selected PBS concentration standard (i.e., 2.5 mg PBS mL^{-1}) containing DMB is shown in Supplementary Figure 5b (top spectrum). Using the set of PBS standards, we established linearity in the responses in the NMR peak areas with the nominal PBS concentration in the standards. Combined with the integrated peak responses of DMB, we showed that the analytically measured PBS concentrations were in very good agreement with the nominal PBS concentrations of the standards (Supplementary Figure 5c; slope of approximately 1).

We estimated the limits of detection (LOD) and limits of quantification (LOQ) (both in units of mg PBS mL^{-1}) for PBS in q-^1H NMR analysis based on the standard deviation of baseline signal noise (σ_{noise}) in a spectrum of pure deuterated chloroform in the chemical shift region of δ = 4.00 - 4.25 ppm (as the PBS peak with the shortest height fell in this region):[3]

$$\mathrm{LOD} = 3 \cdot \sigma_{\mathrm{noise}} \, / \, RF \quad (1)$$

$$\mathrm{LOQ} = 10 \cdot \sigma_{\mathrm{noise}} \, / \, RF \quad (2)$$

where *RF* is the PBS response factor (i.e., the relationship between peak height and PBS amount which we determined by analyzing the PBS standards (= 3212 mg^{-1} PBS mL)). Based on the peak responses of these standards, we determined LOD and LOQ of 3.2 and 10.7 µg PBS mL^{-1} according to Supplementary Equations 1 and 2, respectively. The 1:1 linear

relationship between measured and nominal PBS concentrations and the low LOD and LOQ demonstrate that quantitative ^{1}H NMR allows for selective, accurate, sensitive detection of PBS in deuterated chloroform.

We subsequently developed a Soxhlet extraction method to recover PBS from the same soil that we used in the incubation experiments. To this end, we added 2.5 mg of non-labelled PBS to 2.5 g of soil, thereby obtaining the same PBS concentration in the soil that we used at the start of the actual soil incubation experiments. For the analyses of extraction efficiencies, we added PBS in two different forms: as particles (> 300 μm diameter) or dissolved in chloroform (i.e., 100 μL of a PBS solution spiked onto the soil surface). We included the second type of addition to allow for maximum interactions between dissolved PBS strands and soil particle surfaces after evaporative removal of the chloroform. We subsequently Soxhlet extracted these soils with a chloroform/methanol solvent mixture (90/10 volume%), dried the extract, and then reconstituted the dried extract into deuterated chloroform with DMB as internal standard following a method recently introduced.[4] We obtained complete recovery of both added particulate and dissolved PBS from the soil (i.e., recoveries of 99.3 ± 0.1% and 99.8 ± 0.7%, respectively). The ^{1}H NMR spectra of PBS soils extracts were very clean (Supplementary Figure 5b, bottom spectrum), implying that there was only small interference on PBS quantification from co-extracted SOM.

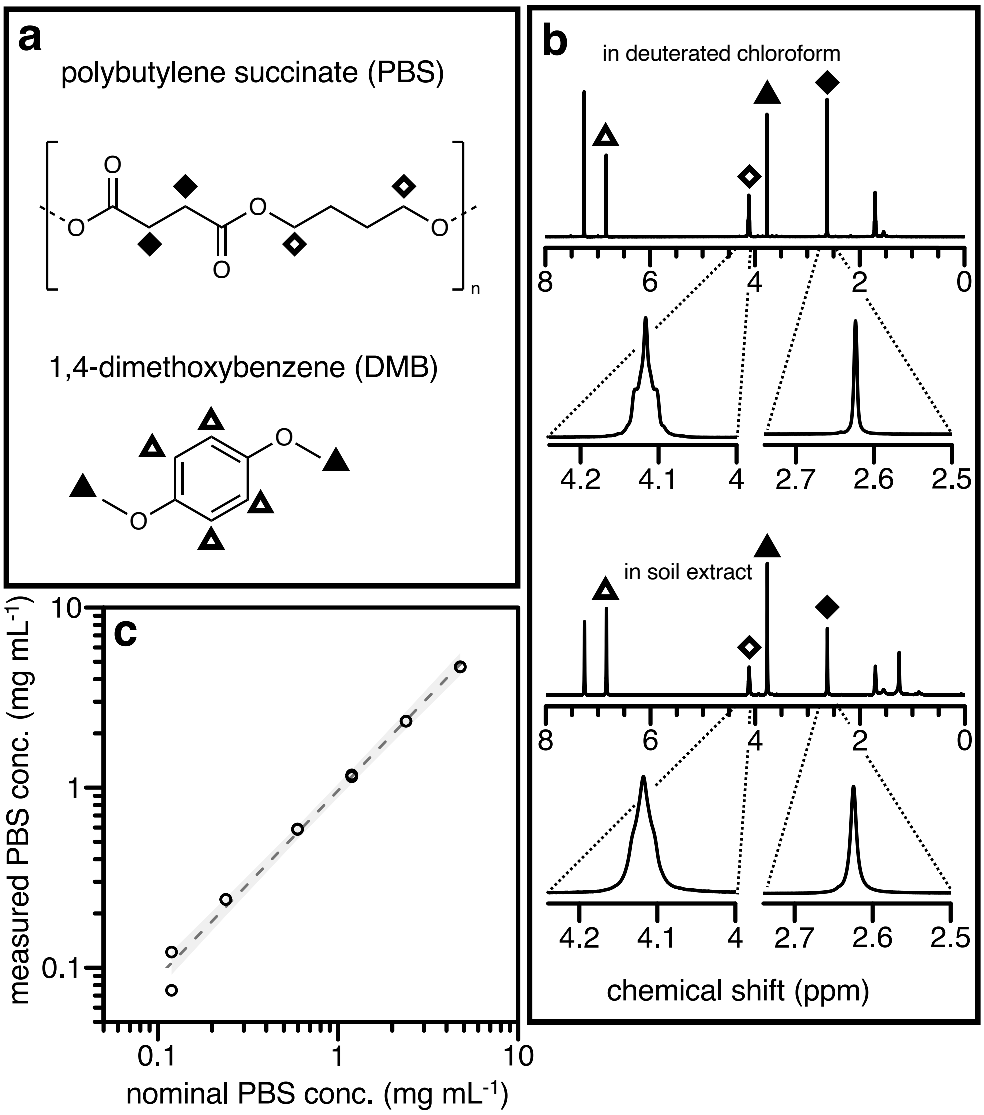

Supplementary Figure 5. Quantification of poly(butylene succinate) (PBS) using quantitative proton nuclear magnetic resonance spectroscopy (q-^{1}H NMR). Panel a: Chemical structures of PBS and of the internal quantification standard 1,4-dimethoxybenzene (DMB). Panel b: ^{1}H NMR spectra of (i) PBS and DMB dissolved in pure deuterated chloroform (upper spectrum), and (ii) PBS and DMB in soil extract (i.e., PBS added to the soil, followed by Soxhlet extracting with chloroform/methanol, complete evaporation of solvent from the extract, re-constituting the dried extract in deuterated chloroform, and DMB addition (lower spectrum)). The symbols mark the positions of protons in PBS (diamonds) and DMB (triangles) and their corresponding peaks in the ^{1}H NMR spectra. We used the sum of the areas under these indicated peaks (normalized to the number of protons per compound) for PBS quantification. Panel c: Linear response in the amounts of PBS quantified by ^{1}H NMR to PBS concentration in standards prepared with DMB as internal quantification standard. Duplicate standards were prepared at each PBS concentration (i.e., six concentrations resulting in a total of 12 standards) with each standard being analyzed once; points are shown for individual standards, which in most cases overlap. All data was included in fitting. The dashed line is the linear regression fit and the shaded area depicts the 95% confidence interval of the fit (calculated slope ± 95% confidence interval = 1.04 ± 0.03; calculated intercept ± 95% confidence interval = -0.04 ± 0.04 mg PBS mL^{-1}; R^2 = 0.992).

Supplementary Note 6

Compilation of $C_{mineralized}$*,* $C_{non\text{-}mineralized}$*, and* $C_{polymer\ residual}$ *data for all incubations*

Supplementary Table 1 compiles the $C_{mineralized}$, $C_{non\text{-}mineralized}$, and $C_{polymer\ residual}$ data for each PBS and cellulose incubation experiment.

Supplementary Table 1. Compilation of poly(butylene succinate) (PBS) and cellulose biodegradation results for all triplicate soil incubations.

polymer	replicate #	incubation time (d)	$C_{mineralized}$ [a]	$C_{non\text{-}mineralized}$ [b]	$C_{polymer\ residual}$ [c]
			(% of added polymer-^{13}C)		
Poly(butylene succinate) (PBS)					
	1	319	49.7	44.7	50.8 (± 1.1)
PB(1,4-$^{13}C_2$-S)	2	425	64.8	34.0	34.9 (± 1.5)
	3	425	64.6	35.4	38.9 (± 1.3)
	1	319	66.6	35.1	26.6 (± 0.9)
PB(2,3-$^{13}C_2$-S)	2	425	68.4	32.1	24.8 (± 1.0)
	3	425	66.3	32.3	27.6 (± 1.9)
	1	319	60.1	40.0	35.8 (± 1.4)
P($^{13}C_4$-B)S	2	425	63.6	36.2	28.4 (± 2.7)
	3	425	59.6	36.7	29.2 (± 0.3)
Cellulose					
	1	139	73.6	23.5	N/A
U-$^{13}C_6$-cellulose	2	254	75.9	22.3	N/A
	3	254	75.3	22.3	N/A

[a] data also plotted in main text in Figures 1b and 3 for PBS and Figures 1f and 3 for cellulose; cumulative mineralization at the end of incubation time per bottle

[b] data is plotted in main text in Figure 3; a single EA-IRMS analysis was carried out for each soil taken from the respective incubation bottle

[c] data also plotted in main text in Figure 4 as percent of $C_{non\text{-}mineralized}$; three replicate extractions were performed for each soil from the respective incubation bottle (reported as mean extracted amount ± standard deviation).

Supplementary Note 7

Kinetic modeling of polymer biodegradation in soils

I. Model equations

Using the box model shown in the Methods section of the main text, we set up the carbon flux model for polymer and monomer biodegradation data by defining the carbon flux between different pools with the following ordinary differential equations in COPASI.

For cellulose, we assumed the depolymerization to be first order with respect to the amount of cellulose remaining, $C_{cellulose}$ (μg C):

$$\frac{\mathrm{d}\,\mathrm{C}_{\mathrm{cellulose}}}{\mathrm{d}\,t} = -k_1 \cdot F_{\mathrm{colonized}} \cdot \mathrm{C}_{\mathrm{cellulose}} \qquad (3)$$

where k_1 is the depolymerization rate constant (h^{-1}) and $F_{colonized}$ represents the fraction of the polymer surface which is accessible for enzymatic cellulose hydrolysis (see below for more details).

For PBS, we slightly modified this rate equation. We added PBS to soils in the form of small particles (100–300 μm diameter, obtained by cryomilling the synthesized bulk PBS material) and assumed that hydrolytic breakdown (by extracellular microbial esterases) only occurred on the particle surfaces. For this reason, we set the depolymerization rate to be two-thirds order with respect to the amount of PBS remaining, C_{PBS} (μg C). This modification was based on the assumption that PBS particles were spherical, and that continuous hydrolysis progressively decreased the radius of the PBS particles. In this case, the surface area of a sphere, SA_S (cm^2), depends on the sphere volume, V_S (cm^3), according to:

$$SA_{\mathrm{S}} = 3/r \cdot V_{\mathrm{S}}^{(2/3)} \qquad (4)$$

Where r (cm) is the radius of the sphere.

The PBS depolymerization rate equation is then given as:

$$\frac{\mathrm{d}\,\mathrm{C}_{\mathrm{PBS}}}{\mathrm{d}\,t} = -k_1 \cdot F_{\mathrm{colonized}} \cdot \mathrm{C}_{\mathrm{PBS}}^{\ (2/3)} \qquad (5)$$

where C_{PBS} (µg C) is the amount of PBS remaining at any given time.

We fitted the biodegradation data of PBS in two ways. First, we fitted the data using a single and constant value for k_1 over the course of the incubation. Second, we allowed for a decrease in the effective PBS breakdown rate constant k'_1 (h^{-1}) by multiplying k_1 (h^{-1}) with a factor that exponentially decayed from unity over time:

$$k'_1 = k_1 \cdot e^{-m_1 \cdot t} \tag{6}$$

where m_1 (h^{-1}) is a fitted kinetic parameter that describes the decrease in the effective hydrolytic breakdown rate constant of PBS and t (h) is the time during the incubation.

Both PBS and cellulose showed an initial lag phase in mineralization following their addition to the soil (i.e., the mineralization curves showed an upward curvature during the first days of the incubations). We ascribe this lag phase to the initial time needed for microbial colonization of the PBS and cellulose surfaces and the secretion of microbial extracellular hydrolases. We modeled this lag phase by the term $F_{colonized}$ by allowing it to increase from 0 to unity according to the following function:

$$F_{colonized} = 1 - e^{-m_2 \cdot t} \tag{7}$$

where m_2 is a fitted kinetic rate constant (h^{-1}) that describes the overall rate at which $F_{colonized}$ increased over time.

The carbon from the polymers, $C_{polymer}$, was hydrolytically broken down into low molecular weight products (monomers and short oligomers) that we considered to be readily utilizable by the soil microbes (i.e., labile carbon, C_{labile}). We modeled uptake and utilization with a first order rate law with respect to C_{labile} (µg C) and a rate constant k_2 (h^{-1}). The rate of change of the size of the C_{labile} pool was therefore described the following equation that accounted for both the input to and output of carbon from this pool:

$$\frac{d\,C_{labile}}{d\,t} = \left(k_1 \cdot F_{colonized} \cdot C_{polymer}^{\gamma}\right) - \left(k_2 \cdot C_{labile}\right) \tag{8}$$

where $\gamma = 1$ for $C_{cellulose}$ and $\gamma = 2/3$ for C_{PBS}.

Microorganisms utilize the carbon in C_{labile} both catabolically under formation of CO_2 (i.e., direct transfer of carbon to $C_{mineralized}$) and anabolically to form new biomass (i.e., transfer of carbon to $C_{biomass}$). The fraction of utilized carbon which was incorporated into biomass is defined as the carbon use efficiency (CUE). Finally, we also allowed that PBS- and cellulose-derived carbon in $C_{biomass}$ became mineralized, according to a first order rate law with respect to $C_{biomass}$ and the rate constant k_3 (h^{-1}).

Overall, the carbon fluxes through $C_{biomass}$, $C_{mineralized\ from\ polymer}$, and $C_{mineralized\ from\ biomass}$ were then defined as:

$$\frac{\mathrm{d}\,C_{\mathrm{biomass}}}{\mathrm{d}\,t} = (k_2 \cdot \mathrm{CUE} \cdot C_{\mathrm{labile}}) - (k_3 \cdot C_{\mathrm{biomass}}) \qquad (9)$$

$$\frac{\mathrm{d}\,C_{\mathrm{mineralized\ from\ polymer}}}{\mathrm{d}\,t} = k_2 \cdot (1 - \mathrm{CUE}) \cdot C_{\mathrm{labile}} \qquad (10)$$

$$\frac{\mathrm{d}\,C_{\mathrm{mineralized\ from\ biomass}}}{\mathrm{d}\,t} = k_3 \cdot C_{\mathrm{biomass}} \qquad (11)$$

The total flux into $C_{mineralized}$ was then simply taken as the sum of the two separate sources of $C_{mineralized}$:

$$\frac{\mathrm{d}\,C_{\mathrm{mineralized}}}{\mathrm{d}\,t} = \frac{\mathrm{d}\,C_{\mathrm{mineralized\ from\ polymer}}}{\mathrm{d}\,t} + \frac{\mathrm{d}\,C_{\mathrm{mineralized\ from\ biomass}}}{\mathrm{d}\,t} \qquad (12)$$

For modeling the PBS and cellulose biodegradation data, we set the starting amounts of $C_{polymer}$ to the mass of PBS- and cellulose-^{13}C added (in µg; mean value for triplicates of each PBS variant or of cellulose).

We also modeled the monomer biodegradation data using the same model but omitted the polymer breakdown step (i.e., we directly ascribed the monomers to the C_{labile} pool). We

set the starting amount of C_{labile} to the mass of monomer-^{13}C added to the soils in the incubation experiments (in µg; same value added per triplicate for each labelled monomer).

II. Kinetic parameter estimation

We simultaneously fitted the data from triplicate incubations to obtain single values of each model parameter for each substrate tested (i.e., each of the three PBS variants, each of the ^{13}C-labelled monomers, and for cellulose). To this end, we imported the experimental data (i.e., $C_{mineralized}$ and for some of the PBS model fits also $C_{polymer\ residual}$) into the "Parameter Estimation" task option of COPASI. We subsequently ascribed the experimental mineralization data to the total $C_{mineralized}$ pool (Supplementary Equation 12) in the model. For long-term incubations of PBS and cellulose, we selected a subset of the total mineralization measurement points that we collected with a high measurement frequency during the early phase of the incubations (up to 70 days for cellulose and 60 days for PBS) to allow for a more even distribution of measurement data across the entire incubation period. This procedure ensured that model fits were not biased by the more-frequently collected data during the initial phase of incubations. In Supplementary Table 2 we list the start values and limits for each of the fitted model parameters for the different incubations.

Supplementary Table 2. Start values and limits for parameters fitted by the kinetic biodegradation models

Model parameter	Substrate used in experiment: monomers			cellulose			PBS		
	L.L. [a]	start [b]	U.L. [c]	L.L.	start	U.L.	L.L.	start	U.L.
k_1 (h^{-1})	N/A	N/A	N/A	10^{-6}	10^{-2}	10^{+6}	10^{-6}	$5 \cdot 10^{-3}$	10^{+6}
k_2 (h^{-1})	10^{-6}	10^{-2}	10^{+6}	10^{-6}	10^{-2}	10^{+6}	N/A	d	N/A
k_3 (h^{-1})	10^{-6}	10^{-4}	10^{+6}	10^{-6}	10^{-4}	10^{+6}	10^{-6}	$1 \cdot 10^{-4}$	10^{+6}
m_1 (h^{-1})	N/A	N/A	N/A	N/A	N/A	N/A	10^{-6}	$5 \cdot 10^{-4}$	10^{+6}
m_2 (h^{-1})	N/A	N/A	N/A	10^{-6}	10^{-2}	10^{+6}	10^{-2}	10^{-2}	10^{+6}
CUE	0.01	0.5	1.00	0.01	0.1	0.60	10^{-6}	0.10	0.40

[a] L.L. = lower limit set for the selected parameter
[b] start = the start value used for the parameter optimization
[c] U.L. = upper limit set for the selected parameter

[d] k_2 in each PBS fit was set to the k_2 value that was fitted for the mineralization data of the respective monomer

We fitted the data using the Levenberg-Marquardt optimization method with an iteration limit of 10,000 steps and a tolerance level of 10^{-6} (i.e., the optimization stopped when the difference in the objective function value between two iteration steps was smaller than the tolerance level).

III. Modeling fluxes between carbon pools

Using the fitted model parameters, we calculated modeled amounts of carbon in each pool over the incubation time in each individual experiment. These calculations were performed using the "Time Course" task of COPASI, with the number of time steps set to the maximum total incubation time for each substrate (i.e., 336 hour-time steps for monomers, 254 day-time steps for cellulose, and 425 day-time steps for PBS). This calculated model data was then plotted together with the experimental data (Supplementary Figures 6-9, and Figure 5 of the main manuscript).

IV. Kinetic modeling results

Fitting of monomer data. We first fitted the monomer biodegradation data because biodegradation of these compounds required no hydrolytic breakdown step. Supplementary Table 3 lists the optimized parameters for model fits to each of the three labelled monomers. We note that the rate constant for turnover of $C_{biomass}$ (k_3) was comparable for all three monomers, as expected even though the carbon was microbially utilized in a monomer- and position specific manner.

Supplementary Table 3. Optimized model parameters obtained by fitting the mineralization data of ^{13}C-labelled monomers 1,4-$^{13}C_2$-succinate (S), 2,3-$^{13}C_2$-succinate (S), and $^{13}C_4$-butanediol (B).

	Fitted model parameters			
Monomer	k_2 (h^{-1})	k_3 (h^{-1})	CUE	RMSE
1,4-$^{13}C_2$-S	0.61	$8.6 \cdot 10^{-4}$	0.12	4.08

2,3-$^{13}C_2$-S	0.07	$7.2 \cdot 10^{-4}$	0.88	1.13
$^{13}C_4$-B	0.13	$8.5 \cdot 10^{-4}$	0.66	3.60

The changes in the amount of carbon from the monomers in the different carbon pools over time, calculated based on the optimized model parameters, are shown in Supplementary Figure 6, along with experimental mineralization data of the monomer-^{13}C.

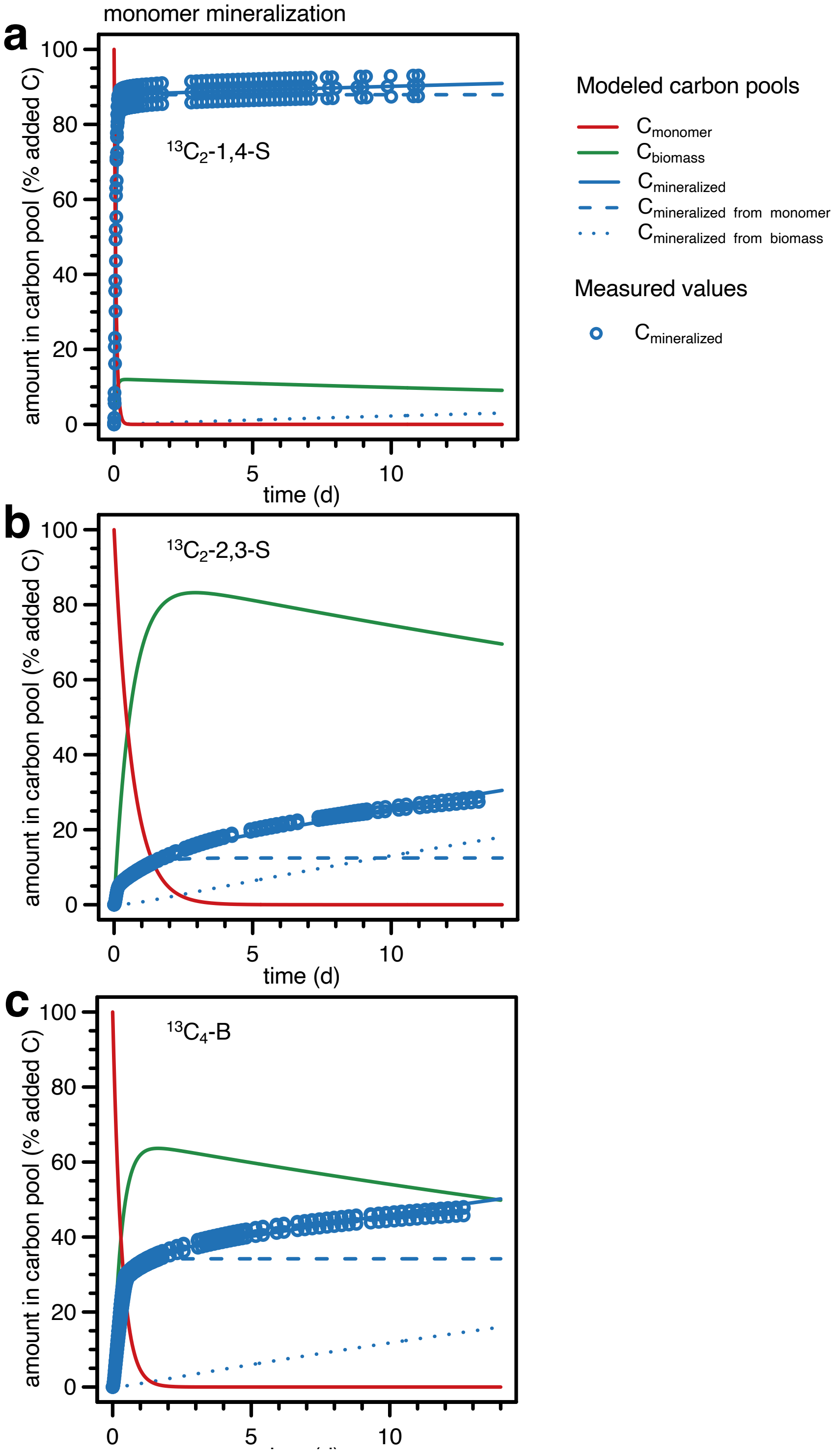

Supplementary Figure 6. Results of kinetic model fit to monomer mineralization data. Modeled carbon pools (i.e., $C_{monomer}$ (red solid line), $C_{biomass}$ (green solid line), $C_{mineralized}$ (blue solid line), $C_{mineralized\ from\ monomer}$ (blue dashed line), $C_{mineralized\ from\ biomass}$ (blue dotted line)) during biodegradation of ^{13}C-labelled monomers (i.e., 1,4-$^{13}C_2$-succinate (S) (panel a); 2,3-$^{13}C_2$-succinate (S) (panel b); $^{13}C_4$-butanediol (B) (panel c) in soil. The carbon pools were calculated from optimized kinetic parameters (Supplementary Table 3) obtained by fitting the carbon flux model to the mineralization data $C_{mineralized}$ (blue open symbols). Experimental data of triplicate incubations for each monomer was pooled prior to fitting the model.

The model adequately fits the rapid monomer- and position-specific mineralization of the three monomers in the soil. The modelling results are fully consistent with our interpretation that the carboxylate carbons of succinate were readily mineralized to CO_2 (i.e., low CUE and high k_2) while the methylene carbons of the same monomer were preferentially incorporated into $C_{biomass}$ (i.e., high CUE).

Fitting of PBS data. To fit the PBS mineralization data, we set k_2 to the values obtained from fitting the respective monomer mineralization data (Supplementary Table 3). This approach ensured that the model (i) accurately fit rapid microbial utilization of compounds in the C_{labile} pool and, therefore, (ii) eliminated false model outcomes that predicted the formation of a large (and longer lived) C_{labile} pool.

In a first step, we subsequently fitted the model only to the PBS mineralization data (i.e., we ignored $C_{polymer\ residual}$ that we experimentally determined by extracting residual PBS from the soil after terminating the incubations). The optimized model parameters are provided in Supplementary Table 4. Supplementary Figure 7 shows the modeled carbon pools calculated based on the optimized model parameters.

Supplementary Table 4. Optimized model parameters obtained by fitting only the mineralization data of the three ^{13}C-labelled poly(butylene succinate) (PBS) variants (and not including the $C_{polmyer\ residual}$ data).

	(set)	Fitted model parameters				
PBS variant	*k_2 (h^{-1})*	k_1 (h^{-1})	k_3 (h^{-1})	m_2 (h^{-1})	CUE	RMSE
PB(1,4-$^{13}C_2$-S)	*0.61*	$4.0 \cdot 10^{-3}$	$2.2 \cdot 10^{-5}$	$1.0 \cdot 10^{-3}$	0.28	66.91
PB(2,3-$^{13}C_2$-S)	*0.07*	$3.6 \cdot 10^{-3}$	$0.1 \cdot 10^{-5}$	$1.0 \cdot 10^{-3}$	0.36	58.01
P($^{13}C_4$-B)S	*0.13*	$4.0 \cdot 10^{-3}$	$1.0 \cdot 10^{-5}$	$1.0 \cdot 10^{-3}$	0.39	34.73

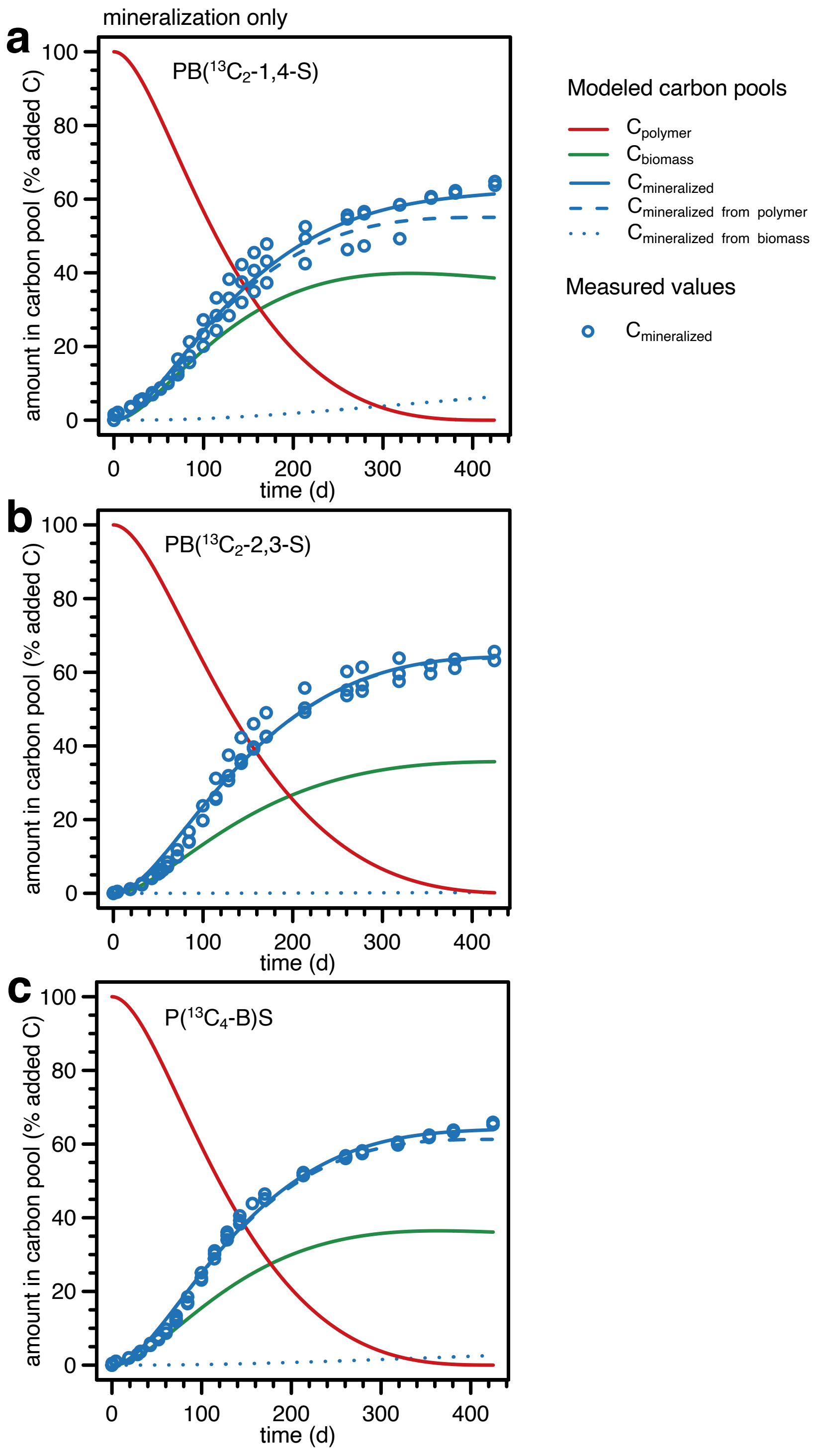

Supplementary Figure 7. Results of kinetic model fit to poly(butylene succinate) (PBS) mineralization data. Modeled carbon pools (i.e., $C_{polymer}$ (red solid line), $C_{biomass}$ (green solid line), $C_{mineralized}$ (blue solid line), $C_{mineralized\ from\ polymer}$ (blue dashed line), $C_{mineralized\ from\ biomass}$ (blue dotted line)) during biodegradation of the three position-specifically ^{13}C-labelled PBS variants (i.e. PB(1,4-$^{13}C_2$-S) (panel a); PB(2,3-$^{13}C_2$-S) (panel b); P($^{13}C_4$-B)S (panel c) in soil. The carbon pools were calculated from optimized kinetic parameters (Supplementary Table 4) obtained by fitting the carbon flux model to only the mineralization data $C_{mineralized}$ (open blue symbols). Experimental data of all (triplicate) incubations for each PBS variant was used for fitting.

While this model adequately described the PBS mineralization data (i.e., $C_{mineralized}$), the optimized model fit falsely predicted complete biodegradation of PBS (i.e., a final $C_{polymer}$ of approximately 0) by the end of the incubations in combination with a large build-up of biomass containing PBS-derived ^{13}C (i.e., $C_{biomass}$ with modeled CUEs = 0.28–0.39). This model run clearly shows that including only the PBS mineralization data in model fitting leads to false model outputs. This result highlights the importance of quantifying $C_{polymer\ residual}$ at the end of the incubations for correct process interpretation.

In a next step, we fitted both $C_{mineralized}$ and $C_{polymer\ residual}$ giving the same weights to the data points for these two pools in the fitting process. The optimized model parameters are given in Supplementary Table 5. Supplementary Figure 8 shows the modeled carbon pools calculated based on the optimized model parameters.

Supplementary Table 5. Optimized model parameters obtained by fitting the data from both the mineralization measurements of the three ^{13}C-labelled poly(butylene succinate (PBS) variants (i.e., $C_{mineralized}$) as well as the residual amounts of the PBS that had remained in the soils at the end of the incubations (i.e., $C_{polymer\ residual}$).

	(set)	Fitted model parameters				
PBS variant	*k_2 (h^{-1})*	k_1 (h^{-1})	k_3 (h^{-1})	m_2 (h^{-1})	CUE	RMSE
PB(1,4-$^{13}C_2$-S)	*0.61*	$1.1 \cdot 10^{-3}$	$2.7 \cdot 10^{-1}$	$2.3 \cdot 10^{-3}$	$1.0 \cdot 10^{-6}$	156.74
PB(2,3-$^{13}C_2$-S)	*0.07*	$1.2 \cdot 10^{-3}$	$1.0 \cdot 10^{-4}$	$2.1 \cdot 10^{-3}$	$3.9 \cdot 10^{-4}$	165.33
P($^{13}C_4$-B)S	*0.13*	$1.2 \cdot 10^{-3}$	$1.2 \cdot 10^{+2}$	$2.4 \cdot 10^{-3}$	$1.0 \cdot 10^{-6}$	155.55

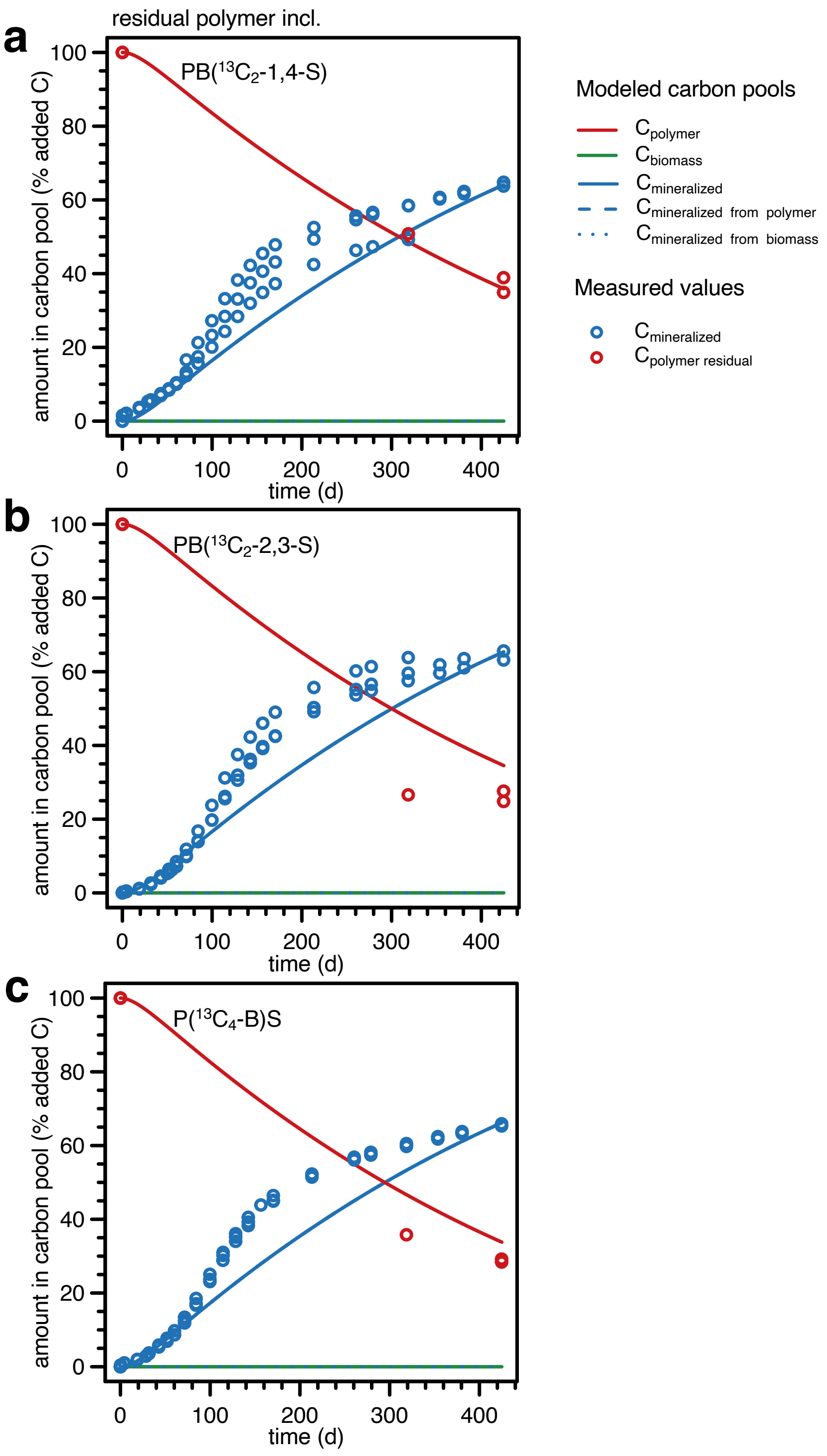

Supplementary Figure 8. **Results of kinetic model fit to poly(butylene succinate) (PBS) biodegradation data, including residual PBS.** Modeled carbon pools (i.e., $C_{polymer}$ (red solid line), $C_{biomass}$ (green solid line), $C_{mineralized}$ (blue solid line), $C_{mineralized\ from\ polymer}$ (blue dashed line), $C_{mineralized\ from\ biomass}$ (blue dotted line)) during biodegradation of position-specifically ^{13}C-labelled PBS variants (i.e. PB(1,4-$^{13}C_2$-S) (panel **a**); PB(2,3-$^{13}C_2$-S) (panel **b**); P($^{13}C_4$-B)S (panel **c**) in soil. The carbon pools were calculated from optimized kinetic parameters (Supplementary Table 5) obtained by fitting the carbon flux model to both the mineralization data $C_{mineralized}$ (open blue symbols) and the extraction data, $C_{polymer\ remaining}$ (open red symbols). Experimental data of all (triplicate) incubations for each PBS variant was used for fitting.

As compared to the model runs in which we fitted only $C_{mineralized}$, the quality of the model fit was lower when we included the extraction data, $C_{polymer\ residual}$, in the fitting process. Supplementary Figure 8 shows that this model could not adequately capture the sigmoidal shape of the $C_{mineralized}$ data with fitting only a single, constant hydrolytic breakdown rate constant k_1. The model run also ascribed $C_{mineralized}$ almost exclusively resulted from direct catabolic utilization of the PBS-carbon with very low CUEs < 0.001.

In a third model, we accounted for an unidentified constraint that started to limit PBS biodegradation during the incubation. The evidence for such a limitation is discussed in the main manuscript. We implemented the limiting constrain into the model mathematically by introducing an exponential decaying factor that is multiplied with the rate constant of PBS hydrolytic breakdown, k_1, to result in a decrease in the 'effective' PBS hydrolytic breakdown rate over the course of the incubation (Supplementary Equation 6). For the model fit shown, we set the CUE to 0.4. However, as shown below in the sensitivity analysis, the experimental data could be well described also with slightly higher (but less realistic) CUE values (tested up to 0.6). The optimized model parameters are provided in Supplementary Table 6. The finding that the data can be fitted with a range of (high) CUE values is consistent with our interpretation that the rate at which PBS-derived carbon cycles through the microbial biomass is larger than the rate at which PBS-derived carbon becomes available to microorganisms. In this case, the buildup of $C_{biomass}$ is only small and can be described by varying CUE values. Supplementary Figure 9 shows the modeled carbon pools calculated based on the optimized model parameters.

Supplementary Table 6. Optimized model parameters obtained by fitting the mineralization and extraction data (i.e., $C_{mineralized}$ and $C_{polymer\ residual}$) of the three ^{13}C-labelled poly(butylene succinate) (PBS) variants. This model included a rate constant m_1 to result in an 'effective' PBS hydrolytic breakdown rate constant that decreased over the course of the incubation.

	(set)	Fitted model parameters					
PBS variant	*k_2 (h^{-1})*	k_1 (h^{-1})	m_1 (h^{-1})	k_3 (h^{-1})	m_2 (h^{-1})	CUE	RMSE
PB(1,4-$^{13}C_2$-S)	*0.61*	$3.7 \cdot 10^{-3}$	$2.5 \cdot 10^{-4}$	$1.9 \cdot 10^{-3}$	$1.0 \cdot 10^{-3}$	0.40	66.47
PB(2,3-$^{13}C_2$-S)	*0.07*	$3.7 \cdot 10^{-3}$	$2.2 \cdot 10^{-4}$	$0.8 \cdot 10^{-3}$	$1.0 \cdot 10^{-3}$	0.40	65.51
P($^{13}C_4$-B)S	*0.13*	$4.0 \cdot 10^{-3}$	$2.4 \cdot 10^{-4}$	$1.0 \cdot 10^{-3}$	$1.0 \cdot 10^{-3}$	0.40	37.67

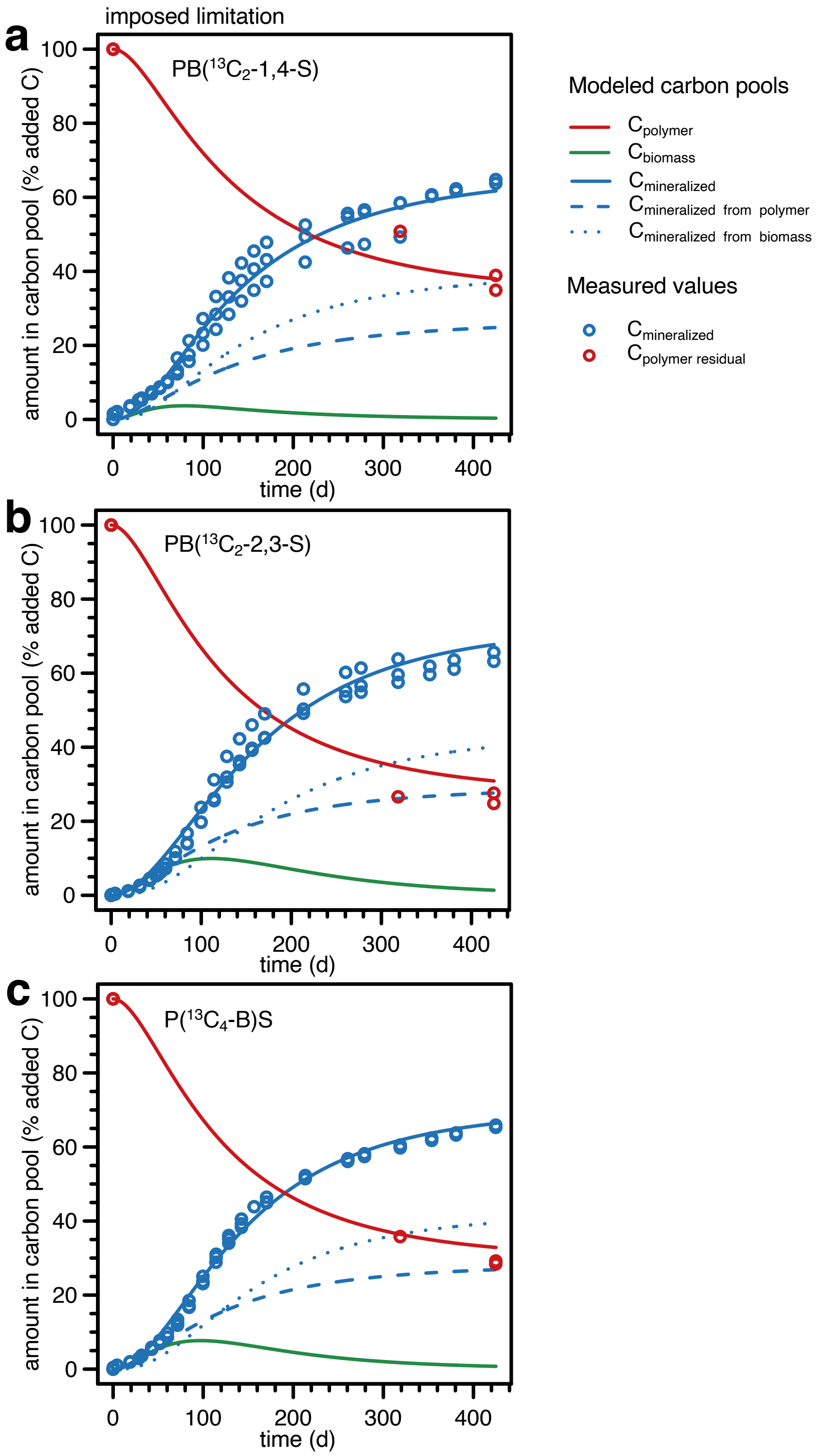

Supplementary Figure 9. Results of kinetic model fit to poly(butylene succinate) (PBS) biodegradation data with an imposed limitation on biodegradation. Modeled carbon pools (i.e., $C_{polymer}$ (red solid line), $C_{biomass}$ (green solid line), $C_{mineralized}$ (blue solid line), $C_{mineralized\ from\ polymer}$ (blue dashed line), $C_{mineralized\ from\ biomass}$ (blue dotted line)) during biodegradation of position-specifically ^{13}C-labelled PBS variants (i.e. PB(1,4-$^{13}C_2$-S) (panel a); PB(2,3-$^{13}C_2$-S) (panel b); P($^{13}C_4$-B)S (panel c) in soil. The carbon pools were calculated from optimized kinetic parameters (Supplementary Table 6) obtained by fitting the carbon flux model to both the

mineralization data $C_{mineralized}$ (open blue symbols) and the extraction data, $C_{polymer\ remaining}$ (open red symbols). In this model, the hydrolytic breakdown rate of PBS was modeled to decrease over time and the upper limit for the fitted carbon use efficiency (CUE) was set to 0.4. Experimental data of all (triplicate) incubations for each PBS variant was used for fitting.

This final model provided a good description of both the mineralization data, $C_{mineralized}$, as well as the extracted residual PBS, $C_{polymer\ residual}$, with RMSEs that were similar to those obtained with the first model run in which residual PBS was not fitted (Supplementary Tables 4 and 6). In this third model, a net buildup of microbial biomass from PBS-derived carbon (i.e., $C_{biomass}$ increases) is predicted up to about 100 days of incubation. Interestingly, up to about 100 days of incubation, $C_{mineralized}$ differed between the three PBS variants (Supplementary Figure 1). We ascribe these differences in the mineralization of the three PBS variants to the monomer- and position-specific utilization of carbon atoms for microbial biomass formation, fully consistent with the fitted net increase in microbial biomass (i.e., variant and monomer-specificity in carbon uptake into microbial biomass was reflected in the $C_{mineralized}$ data as long as $C_{biomass}$ increased). As the incubations progressed, the modeled hydrolytic breakdown rate of PBS decreased over time and, consequently, the rate at which PBS-derived carbon was supplied to the microbial cells became smaller than the rate at which PBS-derived carbon cycled out of the microbial biomass pool through biomass mineralization. Consequently, the modeled size of $C_{biomass}$ pool decreased to a small final pool size at the end of the incubations, consistent with the experimental data (i.e., most of $C_{non\text{-}mineralized}$ was explained by $C_{polymer\ residual}$). We expect that the position-specificity of carbon utilization was kinetically masked by the supply of PBS-derived carbon to the microorganisms (i.e., the supply rate was smaller than the rate at which this carbon cycled through the biomass).

Fitting of cellulose data. We fitted the cellulose mineralization data using the first model described for PBS above and assuming a single, constant depolymerization rate constant k_1. We note that $C_{polymer\ residual}$ was not experimentally accessible for cellulose (i.e., there is no

analytical approach to readily extract cellulose from soils). The optimized model parameters are provided in Supplementary Table 7. Figure 5d in the main text shows the modeled carbon pools calculated based on the optimized model parameters.

Supplementary Table 7. Optimized model parameters obtained by fitting the mineralization data of ^{13}C-labelled cellulose.

	Fitted model parameters					
	k_1 (h^{-1})	k_2 (h^{-1})	k_3 (h^{-1})	m_2 (h^{-1})	CUE	RMSE
cellulose	$1.3 \cdot 10^{-2}$	$5.6 \cdot 10^{-2}$	$8.3 \cdot 10^{-5}$	$4.0 \cdot 10^{-2}$	0.37	104.25

The cellulose mineralization data is well described by the model. We infer from the model output that the biphasic mineralization of cellulose has the following explanations. The first initial rapid mineralization phase reflects direct catabolic utilization of cellulose-^{13}C to form $^{13}CO_2$, while ^{13}C is also incorporated into microbial biomass with a fitted CUE of 0.37 (in agreement with our estimate and published values).[5,6] By contract, the second phase with smaller mineralization rates is ascribed to the mineralization of microbial biomass and SOM that contained cellulose-derived ^{13}C. The outcome of model fit therefore supports our interpretation of the cellulose mineralization provided in the main text.

V. Sensitivity analysis

For the third PBS model above (Supplementary Table 6, Supplementary Figure 9), we conducted a model sensitivity analysis on each of the fitted parameters. For this analysis, we systematically varied one model parameter at a time while all other parameters were fixed to the values provided in Supplementary Table 6 and determined the resulting objective function value. We here present the sensitivity analysis conducted on the data of P($^{13}C_4$-B)S shown in Supplementary Figure 9c and main text Figure 5c (which both show the same model and data). Similar results were obtained for the sensitivity analyses ran on the other two PBS variants. The results of the sensitivity analyses are shown in Supplementary Figure 10 as the change in

the objective function value (a measure for the global quality of fit) vs. the respective model input parameter that we systematically varied.

Supplementary Figure 10 clearly shows that the model was most sensitive to variations in the value of the kinetic parameters that described the PBS hydrolytic breakdown rate (i.e., k_1, m_1, and m_2). The objective function value showed clear and sharp minima when the values of k_1, m_1, or m_2, were varied, and outside of the minima the objective function value was very large (i.e., fits were of poorer quality). By comparison, variations in the rate constant for conversion of $C_{biomass}$ to $C_{mineralized}$, k_3, resulted in a less sharp, but still clear, minimum in the objective function value. The rate constant for the utilization of C_{labile}, k_2, caused no change in the objective function value when it was increased from its optimal value, but a strong change when it was decreased. In this case, we had originally set the values of k_2 to relatively high values based on the results of the modeling of monomer mineralization data. As a result, increasing k_2 showed no effect on the model fit, while decreasing k_2 had a substantial effect Lastly, the model showed very low sensitivity to variations in CUE away from its optimum value. Consistently, the experimental data could be fitted over a range of maximum CUE values, for reasons already discussed above. We note that $C_{mineralized}$ had two distinct contributions: CO_2 produced from catabolic utilization of C_{labile} and CO_2 produced from mineralization of $C_{biomass}$ into which polymer-derived carbon was incorporated. As such, this model is likely insensitive to the value of CUE because as long as the rate of mineralization from the biomass pool is faster than the initial utilization rate, the model does not strictly differentiate between these two sources of CO_2 (the ratio of which is defined by the CUE).

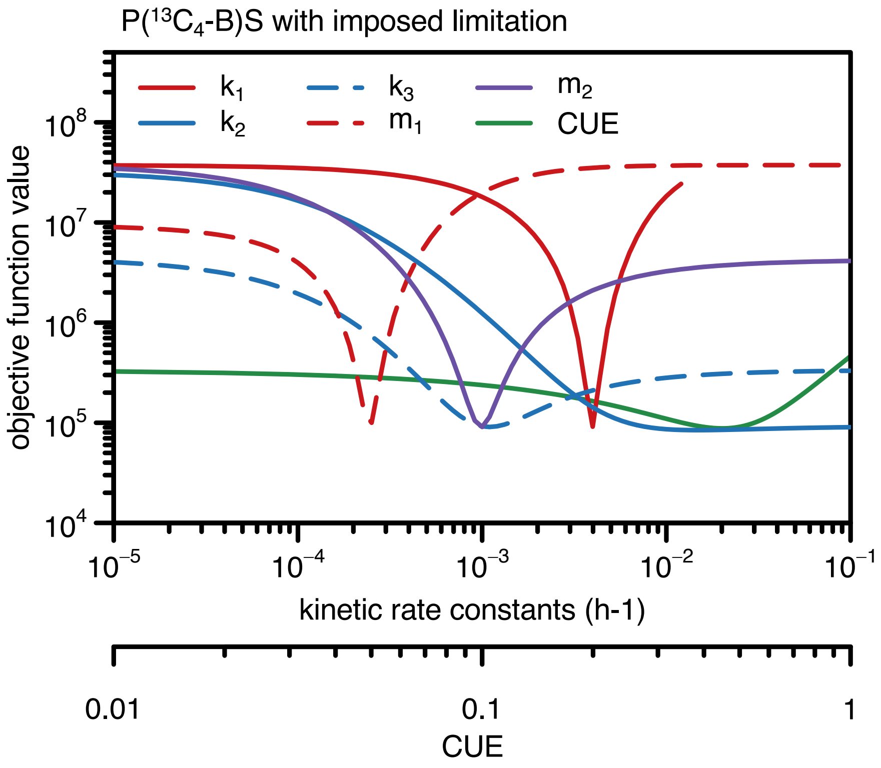

Supplementary Figure 10. Sensitivity analysis of biodegradation kinetic model parameters. Responses of objective function values for model parameter optimization (i.e., metric for goodness-of-fit of modeled results) vs. different values for model parameters (i.e., k_1 (red solid line), k_2 (blue solid line), k_3 (blue dashed line), m_1 (red dashed line), m_2 (purple solid line), and CUE (green solid line)). These curves were obtained by fixing model parameters at their optimized values (Supplementary Table 7) and incrementally varying the one indicated parameter. Model runs were achieved by fitting data from P($^{13}C_4$-B)S using data of $C_{mineralized}$ and $C_{polymer\ residual}$ with a limitation imposed on PBS biodegradation over time (Figure 5c and Supplementary Figure 9c).

Supplementary Note 8

Detailed description of the incubation system

We determined mineralization of ^{13}C-labelled poly(butylene succinate) (PBS) variants, monomers, and cellulose in an automated soil incubation system that we have previously described briefly.[7,8] A schematic of the system is shown in Supplementary Figure 11. At the system inlet, ambient air from the general laboratory ventilation system was continuously pulled into a large-volume (1040 L) intermediate bulk container. This container contained a small fan to continuously mix the air and thereby dampen fluctuations in ambient CO_2 concentrations of the air pulled into the container. Following the container, the air was

humidified by being pulled through the headspace of a series of three closed multi-neck glass bottles, each containing water at the bottom. This air humidification step served to minimize evaporative water losses from the soils in downstream soil incubation bottles. Following the humification chambers, the air was split into 36 individual gas lines, each of which was connected to a single incubation bottle (250 mL Schott glass bottles) containing soils for the incubations. Each incubation bottle was sealed with a custom-made gas tight lid with a gas inlet and outlet. The in- and outlets were positioned relative to each other in a manner that resulted in effective exchange of the headspace in the bottles when gas was pulled through them. Each lid also contained a gas-tight septum to allow for adding water to soils or, in the case of monomer incubations, solutions containing the dissolved monomer to soils without having to open the bottles. Both the air-humidifying bottles and the incubation bottles were housed in a laboratory incubator for accurate temperature control (constant temperature of T= 25.0 ± 0.2 °C) for the entire duration of the experiment. The outlet of each of the 36 incubation bottles was connected to a 3-way, 2-position solenoid valve (type 6122, Burkert). All valves were controlled by an automated logic controller, which actuated one valve at a time to direct the air from the respective incubation bottle to an isotope-sensitive cavity ring-down spectroscopy analyzer (CRDS; model G2201i, Picarro) for real-time quantification of $^{13}CO_2$ and $^{12}CO_2$ concentrations in the efflux air of the bottles at a constant volumetric flow rate of 24 mL min^{-1}. This flow rate was controlled in the CRDS and pumped by a diaphragm pump connected to the CRDS. The headspace air from each of the remaining 35 incubation bottles was continuously exchanged by being pulled through the unactuated solenoid valve to an external diaphragm pump (KNF). Each line from the outlet of the unactuated valve to the diaphragm pump contained a rotameter (model Q-Flow 80, Vögtlin) which was set to a volumetric flow rate of 24 mL min^{-1}. The volumetric flow rate of all bottles being flushed was therefore identical to the volumetric flow rate used in the CRDS gas analysis. The equal flow

rates ensured that the $^{13}CO_2$ and $^{12}CO_2$ concentrations in the headspace of each bottle were always in steady state equilibrium to the flow rate used in CRDS analyses and, therefore, that real-time $^{13}CO_2$ formation rates from the substrates added to soils could be quantified in a given bottle within a few minutes of becoming connected to the CRDS. Periodically, all 36 incubation bottles were in flushing mode and the CRDS was instead connected to one of three CO_2 calibration gases with known concentrations of $^{13}CO_2$ and $^{12}CO_2$ in synthetic air (Pangas; $[CO_2]_{total}$ = 400, 500, 700 ppm (± 1% relative to concentration) and $\delta^{13}CO_2$ = -5.27, -10.76, -10.21 ‰ (respectively, as quantified by gas-chromatography isotope-ratio mass spectrometry (GC-IRMS)). These gases were pulled from three calibration gas cylinders through 1-way, 2-position solenoid valves.

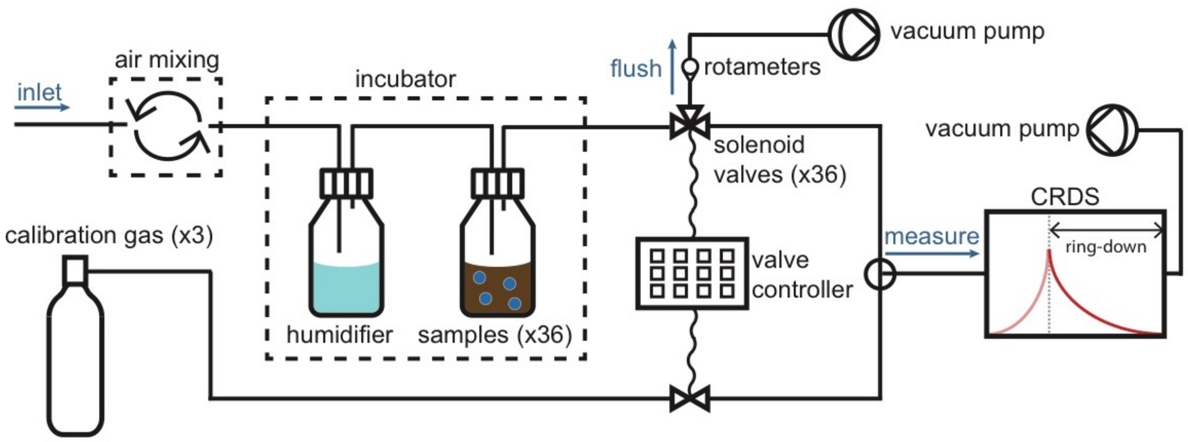

Supplementary Figure 11. Schematic depiction of the automated soil incubation system. This setup was used for the simultaneous quantification of $^{12}CO_2$ and $^{13}CO_2$ formation rates in up to 36 soil incubation bottles using an isotope-sensitive CO_2 cavity ring-down spectroscopy (CRDS) analyzer.

Supplementary References

1. Dunstan, R. H., Whatley, F. R. & Greenaway, W. Growth of *Paracoccus denitrificans* on [2,3-^{13}C]succinate and [1,4-^{13}C]succinate. I. The flux of carbon in energy metabolism and the operation of the TCA cycle. *Proc. R. Soc. Lond. B* **231,** 339–47 (1987).

2. Doelle, H. W. *Bacterial Metabolism*. (Academic Press, 1975).

3. Keith, L. H., Crummett, W., Deegan, J., Libby, R. A., Taylor, J. K. & Wentler, G. Principles of environmental analysis. *Anal. Chem.* **55,** 2210–2218 (1983).

4. Nelson, T. F., Remke, S. C., Kohler, H.-P. E., McNeill, K. & Sander, M. Quantification of synthetic polyesters from biodegradable mulch films in soils. *Environ. Sci. Technol.* **54,** 266–275 (2019).

5. Nicolardot, B., Molina, J. A. E. & Allard, M. R. C and N fluxes between pools of soil organic matter: Model calibration with long-term incubation data. *Soil Biol. Biochem.* **26,** 235–243 (1994).

6. Chiellini, E., Corti, A., D'Antone, S. & Billingham, N. C. Microbial biomass yield and turnover in soil biodegradation tests: carbon substrate effects. *J. Polym. Environ.* **15,** 169–178 (2007).

7. Bai, M., Köstler, M., Kunstmann, J., Wilske, B., Gattinger, A., Frede, H. & Breuer, L. Biodegradability screening of soil amendments through coupling of wavelength-scanned cavity ring-down spectroscopy to multiple dynamic chambers. *Rapid Commun. Mass Spectrom.* **25,** 3683–3689 (2011).

8. Zumstein, M. T., Schintlmeister, A., Nelson, T. F., Baumgartner, R., Woebken, D., Wagner, M., Kohler, H.-P. E., McNeill, K. & Sander, M. Biodegradation of synthetic polymers in soils: tracking carbon into CO_2 and microbial biomass. *Science Advances* **4,** eaas9024 (2018).
