# Supplementary material for: Biodegradation of poly(butylene succinate) in soil laboratory incubations assessed by stable carbon isotope labelling
Source: Nat Commun. 2022 Sep 28;13:5691. doi: 10.1038/s41467-022-33064-8 (PMC9519748; doi:10.1038/s41467-022-33064-8)
Supplement: Supplementary file 2 — Reporting Summary [file 41467_2022_33064_MOESM2_ESM.pdf]

nature portfolio

Corresponding author(s): Michael Sander

Last updated by author(s): Jun 22, 2022

Reporting Summary

Nature Portfolio wishes to improve the reproducibility of the work that we publish. This form provides structure for consistency and transparency in reporting. For further information on Nature Portfolio policies, see our Editorial Policies and the Editorial Policy Checklist.

Statistics

For all statistical analyses, confirm that the following items are present in the figure legend, table legend, main text, or Methods section.

n/a	Confirmed	
☐	☒	The exact sample size (*n*) for each experimental group/condition, given as a discrete number and unit of measurement
☐	☒	A statement on whether measurements were taken from distinct samples or whether the same sample was measured repeatedly
☐	☒	The statistical test(s) used AND whether they are one- or two-sided<br>*Only common tests should be described solely by name; describe more complex techniques in the Methods section.*
☒	☐	A description of all covariates tested
☒	☐	A description of any assumptions or corrections, such as tests of normality and adjustment for multiple comparisons
☐	☒	A full description of the statistical parameters including central tendency (e.g. means) or other basic estimates (e.g. regression coefficient) AND variation (e.g. standard deviation) or associated estimates of uncertainty (e.g. confidence intervals)
☒	☐	For null hypothesis testing, the test statistic (e.g. *F*, *t*, *r*) with confidence intervals, effect sizes, degrees of freedom and *P* value noted<br>*Give P values as exact values whenever suitable.*
☒	☐	For Bayesian analysis, information on the choice of priors and Markov chain Monte Carlo settings
☒	☐	For hierarchical and complex designs, identification of the appropriate level for tests and full reporting of outcomes
☒	☐	Estimates of effect sizes (e.g. Cohen's *d*, Pearson's *r*), indicating how they were calculated

Our web collection on statistics for biologists contains articles on many of the points above.

Software and code

Policy information about availability of computer code

Data collection	TopSpin (version 3.4), Isodat (version 3.0), COPASI (version 4.34)
Data analysis	R (version 4.1.1), RStudio (version 1.4), Excel (version 16.58), MestReNova (version 14.2.3)

For manuscripts utilizing custom algorithms or software that are central to the research but not yet described in published literature, software must be made available to editors and reviewers. We strongly encourage code deposition in a community repository (e.g. GitHub). See the Nature Portfolio guidelines for submitting code & software for further information.

Data

Policy information about availability of data

All manuscripts must include a data availability statement. This statement should provide the following information, where applicable:

- Accession codes, unique identifiers, or web links for publicly available datasets
- A description of any restrictions on data availability
- For clinical datasets or third party data, please ensure that the statement adheres to our policy

Data availability
The data presented in this manuscript and its supplementary information, as well as code files for the biochemical modeling program COPASI used herein, are available from the ETH Zurich Research Collection at https://doi.org/10.3929/ethz-b-000544231.

Field-specific reporting

Please select the one below that is the best fit for your research. If you are not sure, read the appropriate sections before making your selection.

☐ Life sciences ☐ Behavioural & social sciences ☒ Ecological, evolutionary & environmental sciences

For a reference copy of the document with all sections, see nature.com/documents/nr-reporting-summary-flat.pdf

Ecological, evolutionary & environmental sciences study design

All studies must disclose on these points even when the disclosure is negative.

Study description	This study assesses the biodegradation of poly(butylene succinate) (PBS), its monomer units butanediol (B) and succinate (S), as well as cellulose in an agricultural soil. Biodegradation was monitored by using 13C-labelled substrates, which allowed tracking mineralization of the substrates to 13CO2, closing the mass balance on added 13C at the end of the experiments (by soil combustion in an elemental analyzer coupled to isotope ratio mass spectrometry) and by extracting and quantifying residual PBS from the soils. Incubations of PBS, the monomers and cellulose were performed in triplicates. For PBS and the monomers, three different variants were included that contained the 13C-label in different positions of the monomer units. In total, the following samples provided the basis for the work:<br>(i) Three PBS variants, each in triplicate: nine samples<br>(ii) One monomer butanediol and two monomer succinate variants, each in triplicate: nine samples<br>(iii) cellulose in triplicates: three samples.<br>These samples were analyzed for mineralization of substrate carbon to CO2, mass balance at the end of the incubation (for PBS and cellulose) and residual polymer (for PBS).<br>In addition, the work includes additional samples used for method development (e.g., development of an accurate method to quantify residual PBS in the soils).<br>Biodegradation of the added PBS was followed up to 425 days of soil incubation.
Research sample	The three 13C-labelled PBS variants (triplicates each) are the central samples in this study. For the following reason, we decided to incubate triplicates of three PBS variants instead of incubating only a single PBS variant but in a larger number of replicates.<br>First, the three variants varied in the monomer units that were 13C-labelled. This design allowed demonstrating that both monomeric units in PBS (i.e., B and S) undergo biodegradation.<br>Second, we complemented incubations of the three PBS variants with incubations of the corresponding labeled succinate and butanediol, the monomers of PBS, in the same soils. This combination provided strong evidence that PBS hydrolytic breakdown to oligomers and monomers controlled overall biodegradation rates while microbial utilization of the monomeric units in the soil was very fast. Furthermore, mineralization of the monomers was strongly monomer- and carbon-position specific, similar to what we observed in the first days of incubation of the corresponding PBS variants. This finding supports that PBS contained low molecular weight oligomers of B and S (likely including the monomers B and S) that diffused out of the PBS and were readily utilized by soil microorganisms.<br>Third, the use of three PBS variants allowed verifying that the analytical capability to follow mineralization and to quantify non-mineralized PBS-13C was independent of the labeled carbon position in the polymer. This finding demonstrated that the analytical approaches were robust.<br>With a single exception, all data shown in this manuscript has not previously been published. The exception is the mineralization data of butanediol B (Figure 1d in the manuscript) which, up to an incubation time of only 4 days, has been previously presented in the supporting information of another paper from our group (Zumstein, M. T. et al. Biodegradation of synthetic polymers in soils: Tracking carbon into CO2 and microbial biomass. Science Advances 4, eaas9024 (2018)). This has been noted in the figure legend of Figure 1.
Sampling strategy	We chose to run triplicate samples for all substrates added (i.e., PBS, monomers, and cellulose) to allow for basic statistical analyses (mean ± standard deviation). For PBS, we largely extended the data set by including three variants that contained the 13C label in different positions. As a result, a total of nine PBS samples were incubated. Mineralization of all substrates to 13CO2 was continuously measured over the course of the incubations, as detailed in the manuscript. Residual non-mineralized PBS- and cellulose-derived 13C in the soils at the end of the incubations was determined for all incubations on soil aliquots. As detailed in the manuscript, we developed a soil treatment procedure to ensure representative sub-sampling of the soils for this analysis. When using this treatment, EA-IRMS analysis of a single aliquot of soil from each incubation was sufficient to accurately quantify non-mineralized substrate-13C in the soils (i.e., for this analysis, we used three biological replicates and no technical replicates). The closing of mass balances on 13C further supported the high accuracy of this analysis.<br>Finally, all soils from PBS incubations were also extracted for residual PBS at the end of the incubations. For this analysis, we chose to perform three independent extractions of each incubation bottle. These separate extractions demonstrated high accuracy and precision of the extraction of residual PBS from soil in each incubation bottle.
Data collection	The data was collected using commercial software that came with the respective instruments. The mineralization data and the data on residual PBS was collected by TFN and RB. The data on non-mineralized PBS and cellulose 13C in the soils at the end of the incubations was collected by MJ.
Timing and spatial scale	As stated above, mineralization of the substrates in soils to 13CO2 was continuously analyzed over the course of the incubations, as shown in the respective figures. Incubation ran from June 2014 until August 2015 for PBS (maximum 425 days), December 2014 to August 2015 for cellulose (maximum of 254 days), and July 2014 until August 2014 for monomers (with staggered exact start dates, each run for a total maximum of 14 days). Polymer-containing incubations were measured with high frequency (i.e., 3 to 4 times per day) in the beginning stages of the incubations (i.e., first ~60 days) to capture the fast mineralization in this phase with high temporal

resolution. Afterwards, these samples were only periodically measured, which was sufficient to capture the slower mineralization in this phase while allowing for the system to be used for other samples. Similarly, monomer incubations were measured at a high frequency (i.e., 3 to 4 times per hour) in the first phase of the incubations (i.e., first ~30 hours), then less frequently (3 to 4 times per day) for the remainder of the incubations up to 14 days. After terminating the incubations, soils were stored at -20 °C until further analysis. EA-IRMS analyses were performed in November 2015 (cellulose samples) and March 2017 (PBS samples). Extraction of soils and NMR analysis of the extracts were performed in March and October 2017, and July-August 2018. Kinetic modeling was performed in September 2018. All experiments were conducted in the laboratory such that spatial scales of sampling do not apply.

Data exclusions	No data was excluded from the analysis
Reproducibility	We did not attempt to replicate or repeat the entire incubation experiment for three reasons: (i) the very long incubation time of up to 425 days (not including the time for subsequent analyses of non-mineralized 13C and residual PBS) rendered a repetition non-feasible given the time constraints on the project, (ii) the high costs of working with 13C-labelled substrates prohibited repetition (this factor already constrained the number of samples that we could initially run), and (iii) the results of the experiment showed high accuracy of our analyses, closed mass balances on added 13C, as well as high precision of all measurements (mineralization extents, non-mineralized PBS or cellulose, and remaining extractable PBS) between experimental replicates (i.e., high reproducibility among triplicate incubation bottles containing the same labelled PBS variant, cellulose, or PBS monomer).
Randomization	Soil incubation bottles were randomly selected to be used as control incubations (i.e., without having substrates added) or samples incubations (i.e., randomly receiving one of the 13C-labelled substrates).
Blinding	Blinding was not applicable to our study as we worked with well defined, discrete samples. The data collected was always sample-specific and needed to be analyzed and treated as such.

Did the study involve field work? ☐ Yes ☒ No

Reporting for specific materials, systems and methods

We require information from authors about some types of materials, experimental systems and methods used in many studies. Here, indicate whether each material, system or method listed is relevant to your study. If you are not sure if a list item applies to your research, read the appropriate section before selecting a response.

Materials & experimental systems

n/a	Involved in the study
☒	☐ Antibodies
☒	☐ Eukaryotic cell lines
☒	☐ Palaeontology and archaeology
☒	☐ Animals and other organisms
☒	☐ Human research participants
☒	☐ Clinical data
☒	☐ Dual use research of concern

Methods

n/a	Involved in the study
☒	☐ ChIP-seq
☒	☐ Flow cytometry
☒	☐ MRI-based neuroimaging
